# MAPK activity dynamics regulate non-cell autonomous effects of oncogene expression

**DOI:** 10.7554/eLife.60541

**Published:** 2020-09-17

**Authors:** Timothy J Aikin, Amy F Peterson, Michael J Pokrass, Helen R Clark, Sergi Regot

**Affiliations:** 1 Department of Molecular Biology and Genetics, The Johns Hopkins University School of MedicineBaltimoreUnited States; 2 The Biochemistry, Cellular, and Molecular Biology Graduate Program, The Johns Hopkins Universtiy School of MedicineBaltimoreUnited States; 3 Department of Oncology, The Johns Hopkins University School of MedicineBaltimoreUnited States; King's College LondonUnited Kingdom; Fred Hutchinson Cancer Research CenterUnited States

**Keywords:** signaling dynamics, biosensors, live-cell imaging, Human

## Abstract

A large fraction of human cancers contain genetic alterations within the Mitogen Activated Protein Kinase (MAPK) signaling network that promote unpredictable phenotypes. Previous studies have shown that the temporal patterns of MAPK activity (i.e. signaling dynamics) differentially regulate cell behavior. However, the role of signaling dynamics in mediating the effects of cancer driving mutations has not been systematically explored. Here, we show that oncogene expression leads to either pulsatile or sustained ERK activity that correlate with opposing cellular behaviors (i.e. proliferation vs. cell cycle arrest, respectively). Moreover, sustained–but not pulsatile–ERK activity triggers ERK activity waves in unperturbed neighboring cells that depend on the membrane metalloprotease ADAM17 and EGFR activity. Interestingly, the ADAM17-EGFR signaling axis coordinates neighboring cell migration toward oncogenic cells and is required for oncogenic cell extrusion. Overall, our data suggests that the temporal patterns of MAPK activity differentially regulate cell autonomous and non-cell autonomous effects of oncogene expression.

## eLife digest

In animals, the MAPK pathway is a network of genes that helps a cell to detect and then respond to an external signal by switching on or off a specific genetic program. In particular, cells use this pathway to communicate with each other. In an individual cell, the MAPK pathway shows fluctuations in activity over time.

Mutations in the genes belonging to the MAPK pathway are often one of the first events that lead to the emergence of cancers. However, different mutations in the genes of the pathway can have diverse effects on a cell’s behavior: some mutations cause the cell to divide while others make it migrate. Recent research has suggested that these effects may be caused by changes in the pattern of MAPK signaling activity over time.

Here, Aikin et al. used fluorescent markers to document how different MAPK mutations influence the behavior of a human breast cell and its healthy neighbors. The experiments showed that cells with different MAPK mutations behaved in one of two ways: the signaling quickly pulsed between high and low levels of activity, or it remained at a sustained high level. In turn, these two signaling patterns altered cell behavior in different ways. Pulsed signaling led to more cell division, while sustained signaling stopped division and increased migration.

Aikin et al. then examined the effect of the MAPK mutations on neighboring healthy cells. Sustained signaling from the cancerous cell caused a wave of signaling activity in the surrounding cells. This led the healthy cells to divide and migrate toward the cancerous cell, pushing it out of the tissue layer. It is not clear if these changes protect against or promote cancer progression in living tissue. However, these results explain why specific cancer mutations cause different behaviors in cells.

## Introduction

**Figure 1.**
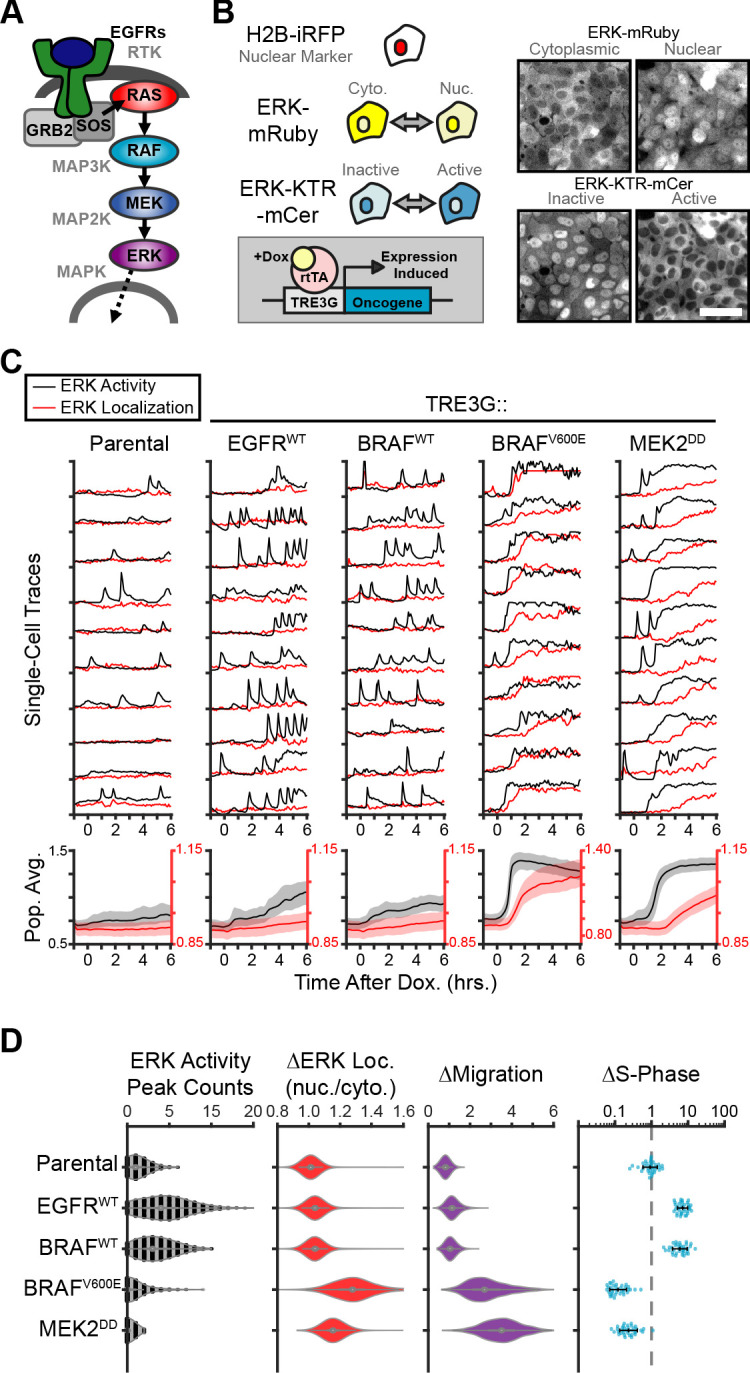
Oncogenic ERK signaling dynamics promote qualitatively different cell behaviors. (**A**) Schematic representation of the RTK/RAS/ERK signaling pathway. (**B**) MCF10A cells were transduced with lentiviral vectors expressing ERK KTR-mCerulean3 and ERK-mRuby2. The doxycycline inducible system (rTtA and TRE3G) was used to drive the expression of oncogenes during live imaging. Representative images of cytoplasmic and nuclear ERK-mRuby2 (top) and inactive or active ERK as reported by ERK KTR-mCerulean3 (bottom). Scale bar = 50 µm. (**C**) Cells described in B with indicated inducible oncogenes were imaged every 5 min for 6 hr upon doxycycline induction (2 µg/ml) at t = 0. Single cells were analyzed as described in methods. Population averages represent more than 1000 cells per condition. Shaded regions indicate the 25^th^-75^th^ percentiles. (**D**) Quantification of data obtained in C. Single-cell counts of ERK activity peaks after induction (6–12 hr), ERK kinase localization fold change (final N/C ratio over basal N/C ratio per cell), and cell migration (final over basal distance traveled per cell) were extracted as described in methods. For proliferation analysis the fraction of S phase cells was measured using Edu incorporation and the change over the no dox control was calculated and normalized to the mean of parental cells (dashed line) (see Materials and methods). Data represents 36 independent observations.

The Receptor-Tyrosine Kinase (RTK)/RAS/ERK signaling axis (Figure 1A) is mutated in most human cancers (Sanchez-Vega et al., 2018). In normal conditions, the ERK pathway promotes proliferation, differentiation, survival and cell migration (Johnson and Lapadat, 2002). During oncogenesis, mutations or amplification of ERK pathway components can also promote oncogene-induced senescence (Hahn and Weinberg, 2002) (OIS) or oncogenic cell extrusion from epithelial monolayers in the so-called Epithelial Defense Against Cancer response (EDAC) (Hogan et al., 2009; Kajita et al., 2010). The mechanisms underlying dose-dependent effects of ERK signaling have been intensely studied using bulk cell population assays. However, the advent of single-cell analysis has shown that single cells often behave qualitatively different than bulk populations. In fact, in vivo and in vitro studies have now shown that pulsatile or sustained ERK activity have different effects on cell behavior (Albeck et al., 2013; Aoki et al., 2013; de la Cova et al., 2017; Johnson and Toettcher, 2019; Santos et al., 2007; Bugaj et al., 2018; Aoki et al., 2017). Whether different oncogenic perturbations also have different functional outcomes depending on downstream signaling dynamics remains unknown. To address this question, an isogenic single-cell approach with temporal control of oncogene expression is needed.

**Figure 1—figure supplement 1.**
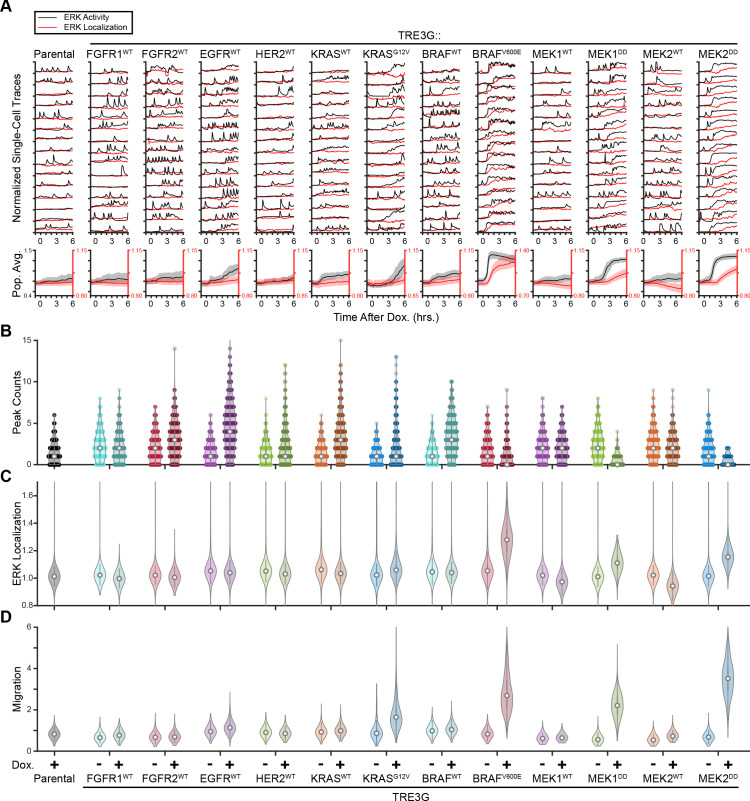
A screen for oncogenic effects on ERK dynamics and cell behavior. (**A**) Inducible cells expressing indicated genes treated and plotted as in 1C. Single traces and population data are reproduced for oncogenes appearing in Figure 1. (**B–D**) Single-cell peaks, ERK nuclear localization, and migration are quantified directly from imaging data as in 1D for indicated inducible cells in the presence or absence of doxycycline (2 µg/ml). Data appearing in Figure 1 is reproduced.

**Figure 1—figure supplement 2.**
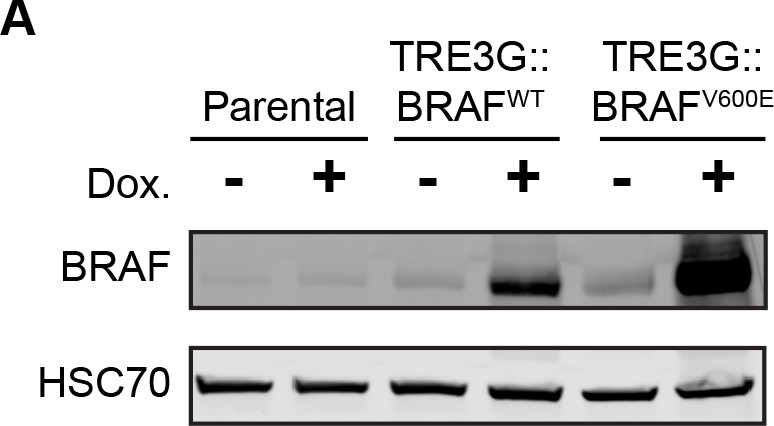
Relative expression of inducible genes. (**A**) Parental, BRAF^WT^, and BRAF^V600E^ inducible cells were grown in the presence or absence of Dox (2 µg/ml) for 24 hr before sample collection and western blot for BRAF and HSC70 (see Materials and methods).

**Figure 1—figure supplement 3.**
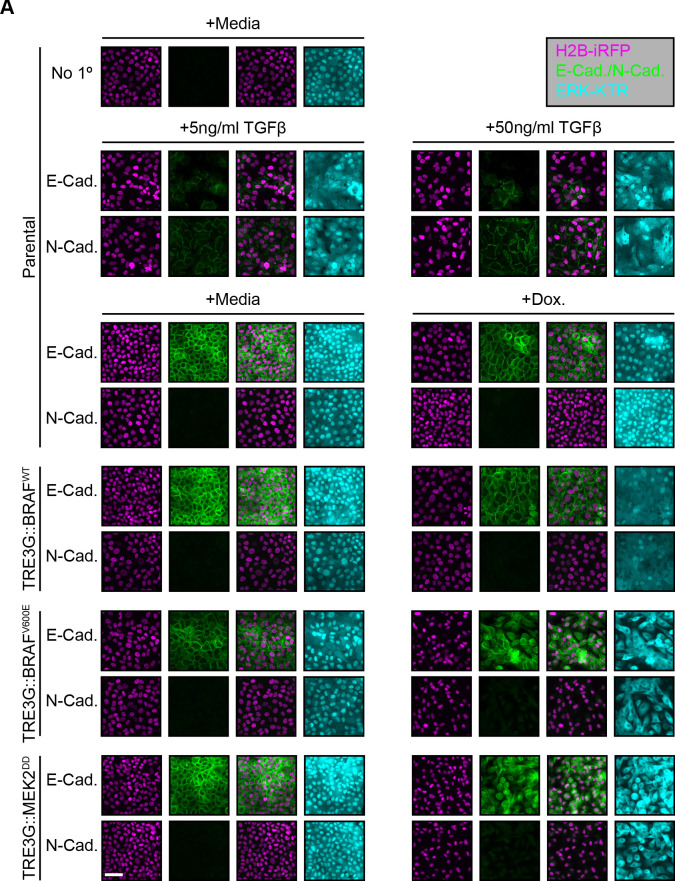
Oncogene-induced cell behaviors are distinct from epithelial-to-mesenchymal transition (EMT). (**A**) Indicated parental or inducible MCF10As were grown in the presence or absence of doxycycline (+Dox., 2 µg/ml) for 24 hr before fixing for immunofluorescence using primary antibodies for E-Cadherin (E-Cad) or N-Cadherin (N-Cad), see Materials and methods. Staining is compared to parental cells treated with TGFβ continuously for several passages over 10 days to induce EMT (5 ng/ml or 50 ng/ml). Nuclear marker (H2B-iRFP, magenta), IF staining (green), an overlay of nuclear marker with IF staining, and ERK activity (ERK-KTR, cyan) are shown. Scale bar = 100 µm.

**Figure 1—video 1.** Different ERK dynamics following oncogene induction. Dual Sensor MCF10A cell lines containing H2B-iRFP nuclear marker (red), ERK-mRuby2 localization reporter (yellow), and an ERK KTR-mCerulean3 representing kinase activity (cyan), and indicated inducible oncogene constructs were treated with doxycycline (2 µg/ml) at time 0 (see Figure 1). Images were collected every 5 min for an hour basal period and 12 hr after induction. Scale bar = 100 µm.

Recent in vivo studies revealed that oncogene expression can trigger tissue level responses involving normal neighboring cells (Brown et al., 2017; Ellis et al., 2019; Clavería et al., 2013; Sancho et al., 2013). In specific cases, mosaic oncogene expression leads to either basal extrusion or apical extrusion (Hogan et al., 2009; Kajita et al., 2010); however, the signaling mechanism responsible for recognition between normal and diseased cells is poorly understood (Kajita and Fujita, 2015; Clavería and Torres, 2016; Maruyama and Fujita, 2017). Coincidentally, propagating ERK signaling waves requiring the sheddase ADAM17 have been observed in mouse epidermis and intestinal organoids, but the physiological role of these signaling events remains unclear (Hiratsuka et al., 2015; Muta et al., 2018). Observation of interactions between oncogenic and neighboring epithelium with live-cell biosensors could provide insights into the collective signaling preceding oncogenic extrusion. In fact, a recent study using live imaging of calcium biosensors during EDAC of HRAS^G12V^ cells showed a calcium signaling wave which propagated through neighboring epithelium to coordinate actin rearrangements and polarized movements during apical extrusion (Takeuchi et al., 2020). The mechanistic basis underlying EDAC calcium waves remains unknown.

Here, we combine live cell imaging of MAPK activity biosensors with inducible expression of oncogenes to study the effects of oncogene expression on signaling dynamics and how altered MAPK dynamics impact both cell autonomous and non-cell autonomous behaviors in epithelial tissues. Our data shows that pulsatile or sustained ERK signaling resulting from oncogenic perturbations triggers different dynamics-dependent cell behaviors including oncogene-induced paracrine signaling via the ADAM17-AREG-EGFR signaling axis. The resulting signaling gradients are required to coordinate neighboring cell migration and active oncogenic cell extrusion (EDAC). Our study highlights the role of MAPK signaling dynamics in coordinating individual and collective cell behaviors.

## Results

To study the effects of oncogene expression on the temporal patterns of MAPK signaling we generated a reporter cell line derived from the chromosomally-normal human breast epithelial line, MCF10A, expressing the ERK Kinase Translocation Reporter (Regot et al., 2014) (ERK KTR) and a fluorescently tagged ERK kinase (ERK-mRuby2). This combination of biosensors allowed independent measurement of ERK activity and ERK localization in live single cells at high temporal resolution. Then, we introduced 12 different doxycycline-inducible oncogenic perturbations via lentiviral infection and measured ERK signaling dynamics during overexpression (Figure 1B). Our results revealed two qualitatively different responses to oncogene induction: (i) increased frequency of ERK activity pulses with no change in ERK kinase localization (i.e. EGFR, B-Raf^WT^), and (ii) sustained ERK activity with subsequent nuclear translocation of ERK kinase (i.e. B-Raf^V600E^, MEK2^DD^) (Figure 1, and Figure 1—Video 1). We refer to these distinct dynamics as pulsatile or sustained ERK, respectively. Of note, MEK1/2^WT^ expression is capable of exporting ERK into the cytoplasm without changing kinase activity (Adachi et al., 2000; Figure 1—figure supplement 1) and ERK nuclear accumulation occurs only when activity is sustained, suggesting that ERK activity and ERK localization are not always correlated. Interestingly, expression of B-Raf^WT^ or B-Raf^V600E^ elicit qualitatively different downstream dynamics even though they differ in a single amino acid and show similar expression levels by immunoblotting (Figure 1—figure supplement 2). Given that the B-Raf^V600E^ is insensitive to negative feedback regulation by ERK (Yao et al., 2015), this result suggests that ERK inhibition to B-Raf^WT^ is mechanistically involved in the characteristic pulsatile dynamics.

Next, we assessed how ERK dynamics affect cell behaviors by measuring cell migration and proliferation. While pulsatile ERK dynamics (i.e. EGFR or B-Raf^WT^) consistently correlated with increased cell cycle progression, sustained ERK activity (i.e. B-Raf^V600E^ or MEK2^DD^) caused cell cycle arrest and increased migration (Figure 1D). Importantly, observed differences in cell behavior correlated with dynamics independently of the point in the cascade that perturbations were introduced (EGFR, Raf or MEK), suggesting that ERK is responsible for differences in cell behaviors rather than alternate downstream pathways. Moreover, expression of B-Raf^WT^ or B-Raf^V600E^, which activate the cascade at the same point, caused different ERK activity dynamics (i.e. pulsatile or sustained respectively) and triggered opposing cellular behaviors (Figure 1D). Taken together, these data suggest that ERK activity dynamics can either promote or inhibit proliferation cell autonomously.

The sudden increase in migration and the loss of cell-cell contacts observed in cases where ERK activity is sustained (B-Raf^V600E^ and MEK2^DD^, Figure 1—Video 1) are reminiscent of phenotypes described for cells undergoing Epithelial-to-Mesenchymal Transition, or EMT (Hao et al., 2019). We sought out to address the role of EMT in oncogene-dependent cell behaviors by immunofluorescent staining of an epithelial marker E-Cadherin (E-Cad) and the mesenchymal marker N-Cadherin (N-Cad). While cell migration was clearly increased at 24 hr post-oncogene expression, cells retain E-Cad expression with no clear increase in N-Cad, as was observed in TGFβ-induced EMT (Figure 1—figure supplement 3). These results indicate that at the time points studied here, altered cell behaviors are either distinct from or precede those resulting from EMT.

**Figure 2.**
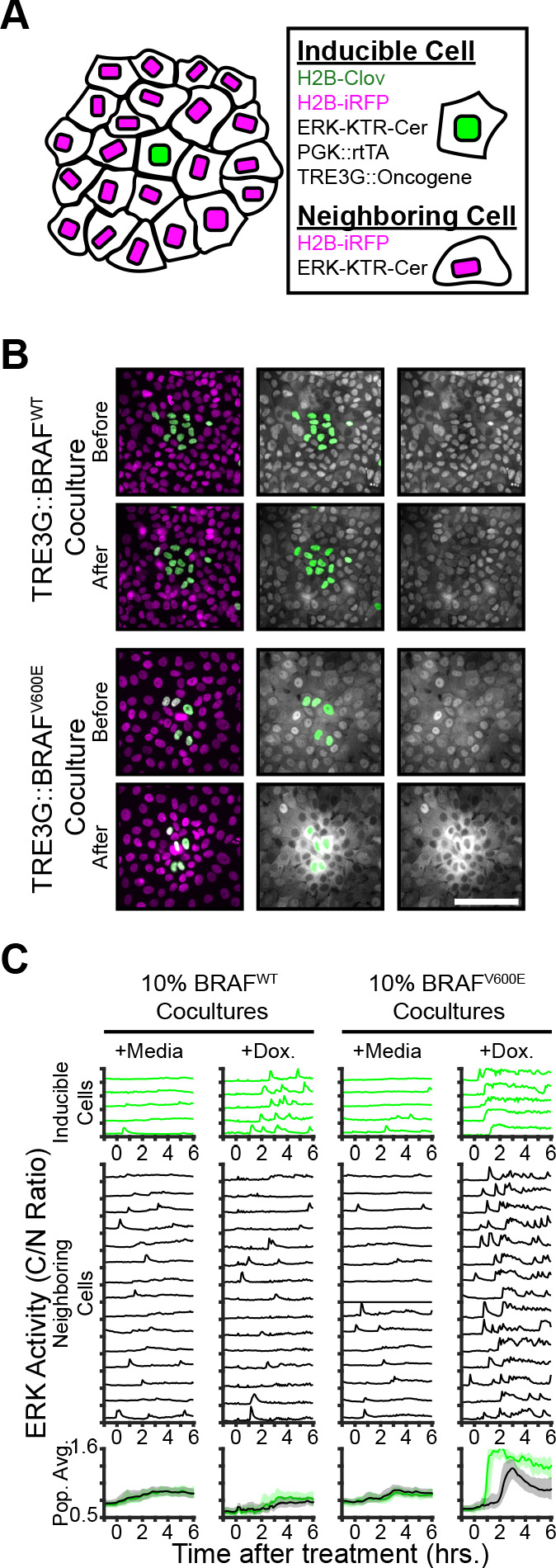
Oncogene induction results in dynamics-dependent paracrine ERK signaling. ( **A**) Schematic representation of coculture assay. H2B-iRFP (magenta) and ERK KTR are expressed in all cells for segmentation and quantification. H2B-mClover (green) was used to label inducible cells. (**B**) BRAF^WT^ or BRAF^V600E^ inducible cells were cocultured at 10% with ERK KTR cells and treated with doxycycline (2 µg/ml). Representative images are shown. Scale bar = 100 µm. (**C**) BRAF^WT^ or BRAF^V600E^ cocultures, as in B, were treated with vehicle (+Media) or with doxycycline (+Dox, 2 µg/ml). Single cells were quantified as described in methods. ERK activity traces in inducible (top, green) and neighboring cells (bottom, black) are shown. Population averages and 25^th^-75^th^ percentiles (shaded) are shown for n > 450 cells per coculture condition.

To examine the non-cell autonomous effects of oncogene expression in epithelial monolayers, we cocultured ‘inducible’ cells (expressing doxycycline-inducible oncogenes, a constitutively expressed H2B-mClover, and the ERK biosensors) with ‘neighboring’ reporter cells (expressing ERK biosensors without inducible oncogenes) and monitored signaling dynamics upon induction (Figure 2A). Interestingly, expression of B-Raf^V600E^, but not B-Raf^WT^, resulted in waves of ERK activation of neighboring cells (Figure 2B–C and Figure 2—Video 1). This comparison suggests that oncogenic perturbations that elicit sustained ERK activity propagate ERK activity pulses to neighboring cells. In agreement, other oncogenes that triggered sustained, but not pulsatile, ERK activity also promoted ERK activity waves in the neighboring cells (Figure 2—figure supplement 1). By using KTRs for p38 and JNK, we observed that neighboring epithelia did not activate other the MAPK pathways (Figure 2—figure supplement 1). Notably, spontaneous cell death events were also followed by similar ERK signaling waves (Figure 2—Video 2), indicating that oncogene expression and cell death may be similarly perceived by neighboring cells.

**Figure 2—figure supplement 1.**
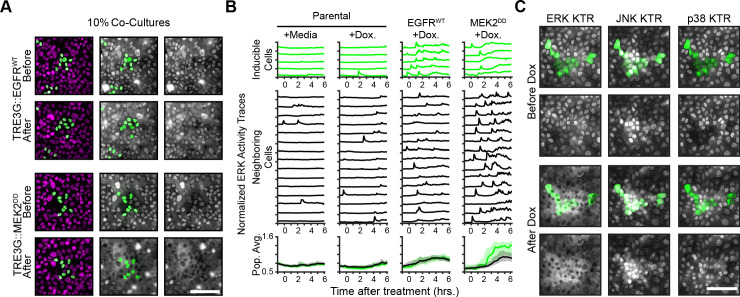
ERK dynamics-dependency of paracrine ERK activation. (**A**) Images from cocultures of inducible EGFR^WT^ and MEK2^DD^ cells presented as in 2B. Scale bar = 100 µm. (**B**) ERK activity traces from cocultures of indicated cells treated as in 2C. Parental cells have an H2B-mClover nuclear marker without any inducible gene system. Population averages and 25^th^-75^th^ percentiles (shaded region) shown for n > 1000 cells. (**C**) The TRE3G-BRAF^V600E^ construct was expressed in a cell line containing ERK KTR-mCerulean3, p38 KTR-mClover, and JNK KTR-mRuby2. These cells were incubated with CellTracker Deep Red dye (green, Invitrogen) and cocultured with unlabeled neighboring cells. Images display the activity of ERK, p38, and JNK before and 6 hr after addition of doxycycline (2 µg/ml). Scale bar = 100 µm.

**Figure 2—video 1.** Oncogene-induced ERK signaling waves. Inducible BRAF^V600E^ cells with a H2B-mClover nuclear marker (green) were cocultured in monolayers with neighboring cells (H2B-iRFP, magenta) (see Figure 2). Images were collected every 5 min for an hour basal period and 12 hr following addition of doxycycline (2 µg/ml) at time 0. ERK KTR-mCer3 (grey) reports ERK activity in all cells. Three examples are shown for both WT (left) and ADAM17^KO^ (right) inducible cells. Scale bar = 100 µm.

**Figure 2—video 2.** Spontaneous cell-death induces ERK signaling waves. Nine examples are shown for spontaneous cell death events in serum-starved MCF10A monolayers imaged every 5 min. The timing of cell death in each example is aligned. ERK KTR-mCer3 represents ERK activity (grey). Scale bar = 100 µm.

**Figure 3.**
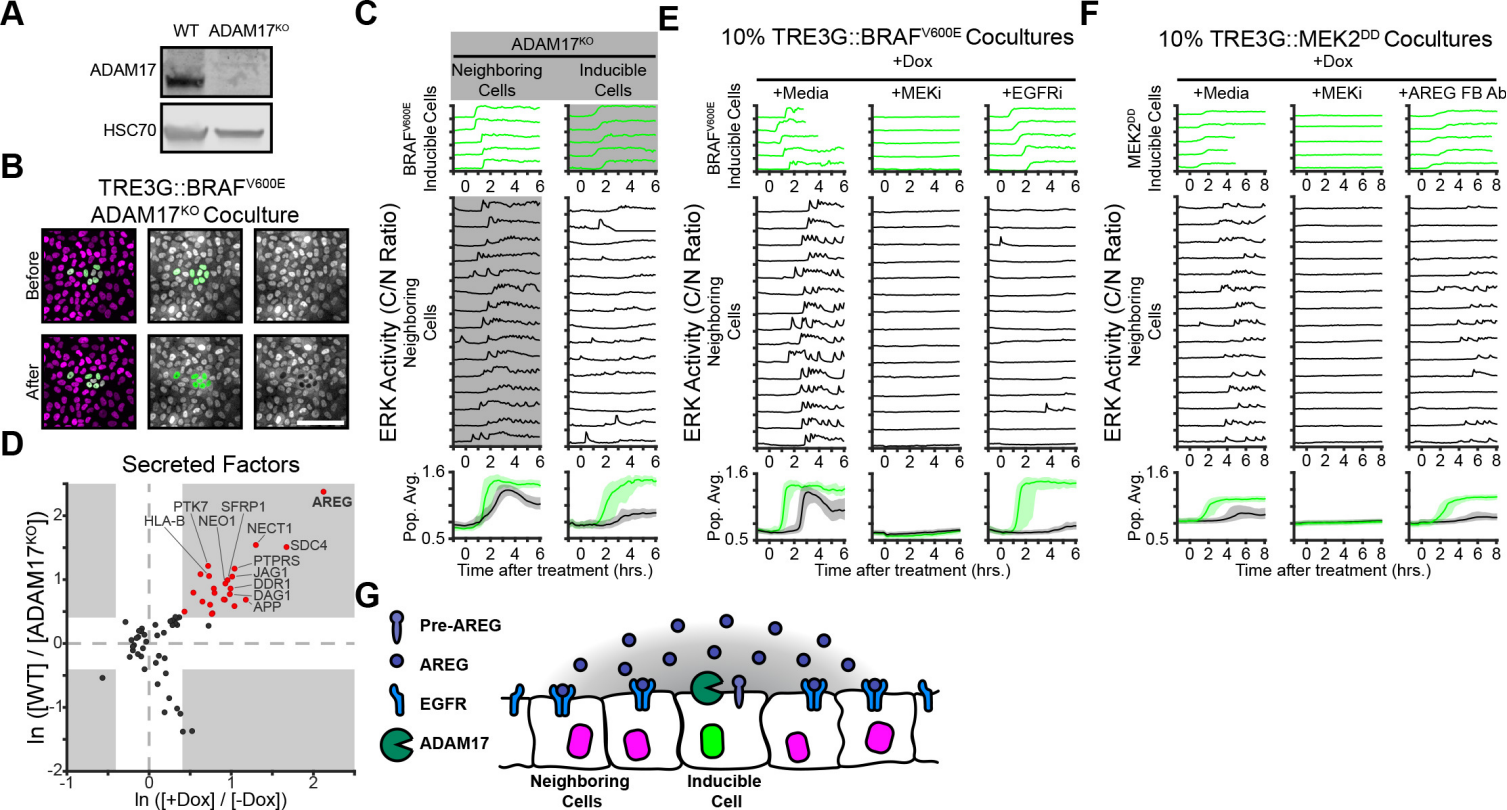
ERK activity waves require ADAM17 release of AREG and neighboring cell EGFRs. (**A**) Immunoblot against ADAM17 and HSC70 in WT and ADAM17^KO^ cells generated by CRISPR-Cas9 editing (see Materials and methods for details). (**B**) Representative images of ADAM17^KO^ BRAF^V600E^ inducible cells cocultured and treated as in Figure 2B. (**C**) ADAM17^KO^ cells (gray boxed traces) were used as inducible cells (right) or neighboring cells (left) in cocultures. Data for n > 1100 cells is presented as in Figure 2C. (**D**) ADAM17 substrates profiled by TMT mass spectrometry. Supernatants from ADAM17^KO^ or WT cells expressing (+Dox) or not expressing (-Dox) BRAF^V600E^ were collected and analyzed by Tandem-Mass-Tag (TMT) mass spectrometry as described in methods. Scatter plots show the natural log of fold change values of all statistically significant (p<0.05) proteins in both WT vs. ADAM17^KO^ and +Dox vs. -Dox comparisons. Grey boxes indicate >1.5 fold change. (**E**) BRAF^V600E^ co-cultured monolayers were plated as in Figure 2C and pretreated with indicated inhibitors (MEKi, 5 µM PD0325901; EGFRi, 5 µM Gefitinib) for one hour before induction with doxycycline (2 µg/ml). Representative single cell traces and population averages for n > 1000 cells are shown as in 2C. (**F**) MEK2^DD^ co-cultured monolayers were plated as in Figure 2C and pretreated with indicated inhibitors (MEKi, 5 µM PD0325901; AREG FB Ab, 50 µg/ml function-blocking antibody) for one hour before induction with doxycycline (2 µg/ml). Representative single cell traces and population averages for n > 1000 cells are shown as in Figure 2C. (**G**) Schematic representation of ADAM17-AREG-EGFR paracrine signaling.

We then addressed the mechanistic basis of oncogene-dependent paracrine signaling. Previous studies demonstrated that ERK waves in epithelial monolayers depend on the membrane-tethered sheddase ADAM17, which releases membrane-bound growth factors that activate EGFR signaling in adjacent cells (Aoki et al., 2013; Aoki et al., 2017; Hiratsuka et al., 2015). Thus, we hypothesized that oncogenic cell ADAM17 may be decoding ERK signaling dynamics to trigger growth factor release. To test this hypothesis, we generated an ADAM17 knockout (ADAM17^KO^) cell line (Figure 3A) and used it as either ‘inducible’ or ‘neighboring’ cells in our coculture assay. Live imaging of WT and ADAM17^KO^ cocultures indicated that ADAM17 is necessary in inducible, but not neighboring cells, to trigger ERK waves in the monolayer (Figure 3B–C and Figure 2—Video 1). Therefore, ADAM17 decodes ERK activity dynamics in inducible cells to transmit ERK signaling to neighboring cells. Previous work has shown that ADAM17 is weakly phosphorylated compared to other ERK substrates (Díaz-Rodríguez et al., 2002), thus the phosphorylation-dephosphorylation kinetics of ADAM17 and the temporal patterns of ERK activity may explain dynamics-specific ADAM17 activation.

ADAM17-released growth factors include HB-EGF, TGF-α, Epiregulin, and Amphiregulin (Zunke and Rose-John, 2017; Rios-Doria et al., 2015). In order to identify the factors mediating oncogene-induced paracrine signaling we used Tandem-Mass-Tag Mass Spectrometry of supernatant proteins following induction of sustained ERK activity in WT and ADAM17^KO^ cells. A variety of known and unknown ADAM17 substrates were present in the induced cell supernatants, including immune surveillance (HLA-A/B/C), Delta-Notch (JAG1), and Wnt (SFRP) signaling proteins (Figure 3D and Supplementary file 1). Of note, the EGFR ligand Amphiregulin (AREG) was the most upregulated, ADAM17-dependent protein in the supernatant, suggesting that AREG released from inducible cells could act as an oncogene-dependent paracrine signaling molecule. Accordingly, cocultures pre-incubated with AREG function-blocking antibodies or EGFR inhibitors prevented neighboring cell ERK activation without affecting ERK signaling in inducible cells (Figure 3E–F). These results indicate that oncogene-dependent ERK waves are mediated by ADAM17 (in inducible cells), AREG release, and EGFR signaling (Figure 3G).

**Figure 4.**
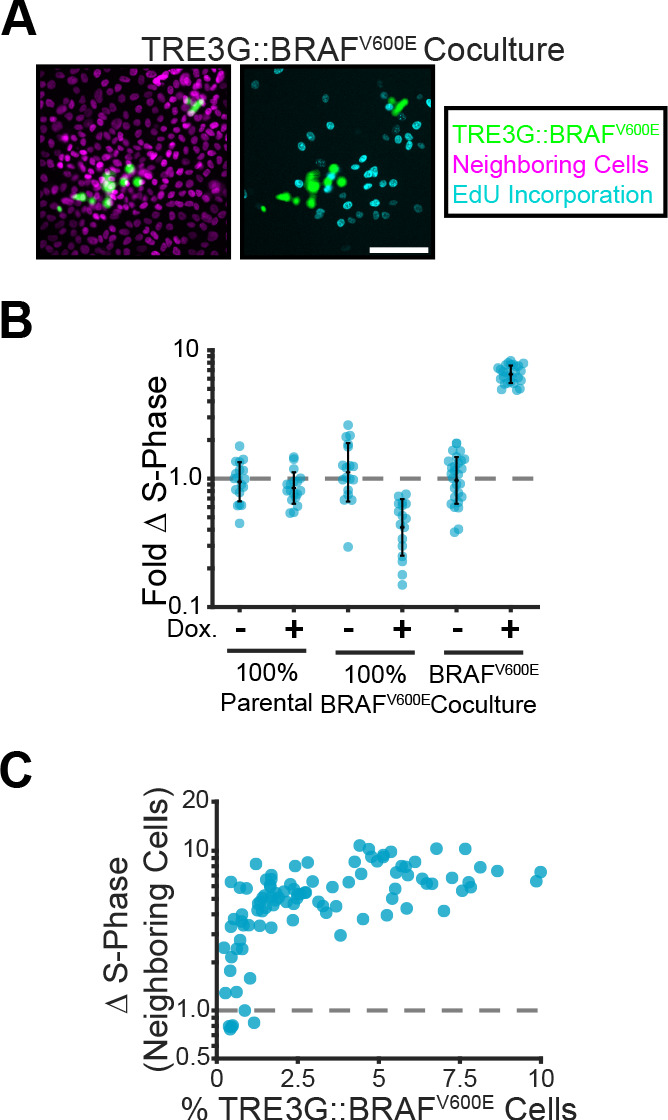
Paracrine ERK signaling leads to non-cell autonomous proliferation. (**A**) Representative images of BRAF^V600E^ cocultures treated with doxycycline and EdU as described in methods. Inducible cell nuclei (H2B-mClover, green), all nuclei (H2B-iRFP, magenta) and EdU staining (cyan) are shown. Scale bar = 100 µm. (**B**) Indicated monolayers were cultured and incubated with or without doxycycline for 24 hr. The change in S-phase cell fractions was determined by EdU incorporation as described in methods and normalized to parental mean (dashed line). Bar represents mean and standard deviation for n ≥﻿ 16 observations. (**C**) Inducible BRAF^V600E^ cocultures were plated at different proportions and labelled with EdU as in A. The fold-change in S-phase cell fractions is plotted against the percent of BRAF^V600E^-expressing cells for each position. 98 total observations shown.

Given that cells surrounding B-Raf^V600E^ expressing cells showed pulsatile ERK activity (Figure 2C), we hypothesized that oncogene expression may promote cell proliferation in a non-cell autonomous manner. Accordingly, sustained ERK signaling in inducible cells increased proliferation of neighboring cells up to 10-fold (Figure 4A–C). Together, these data indicate that, depending on ERK dynamics, oncogenic cells can have either cell autonomous or non-cell autonomous contributions to tissue growth.

**Figure 5.**
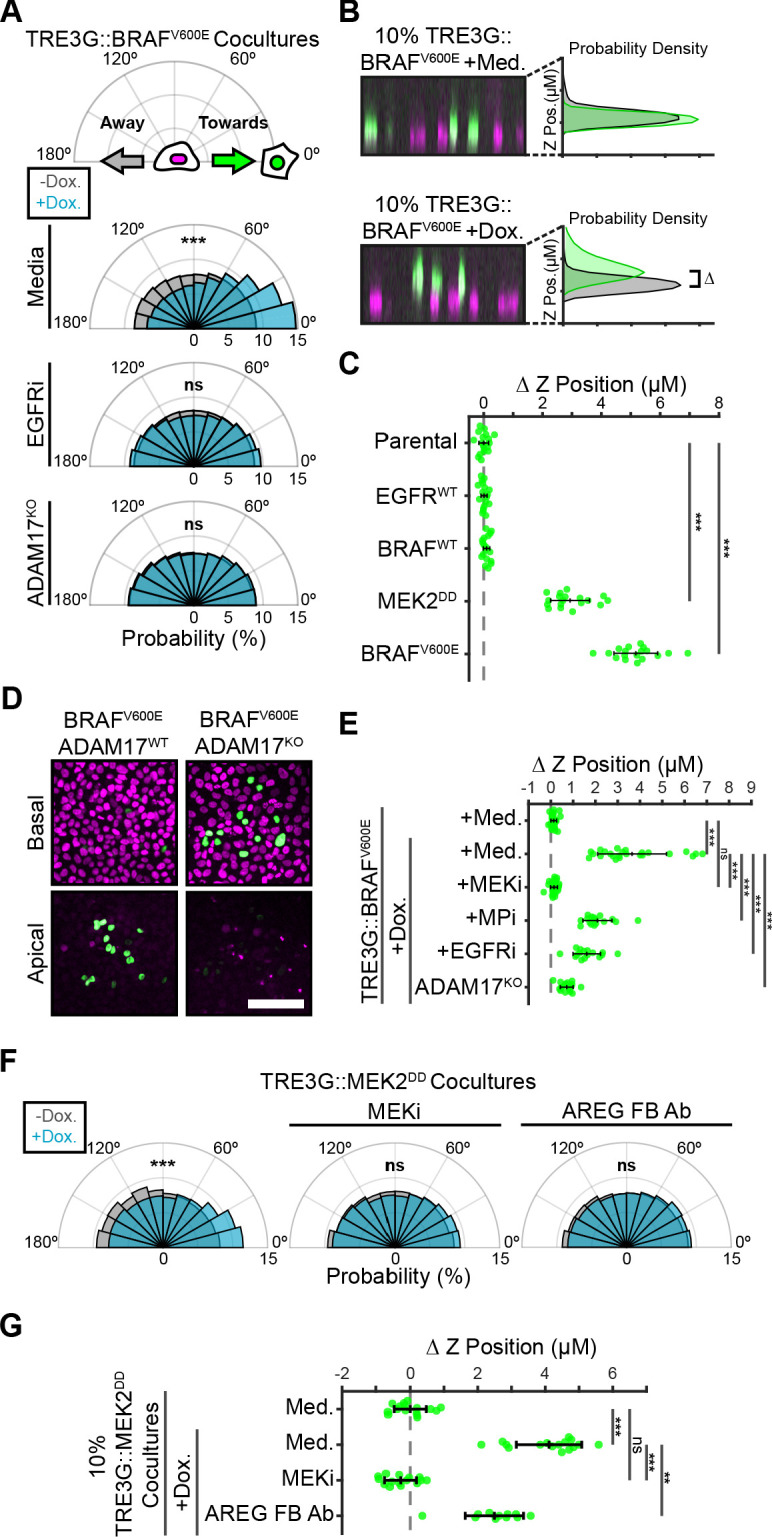
Paracrine ERK activation coordinates extrusion of aberrantly signaling cells through directed migration of the neighboring epithelium. (**A**) Inducible BRAF^V600E^ cells (WT or ADAM17^KO^) were plated in 1% cocultures and treated with doxycycline (2 µg/ml) in the presence or absence of EGFR inhibitor gefitinib (5 µM) as indicated. Radial histograms represent migration angle distributions of neighboring cells before (grey) and 2–6 hr after (cyan) induction (see Materials and methods). Data represents angles from n > 1000 cells from 10 independent observations per condition. Data was assessed using subsampling and a two-sample KS test with ‘ns’ not significant, ***p<0.001 (see Materials and methods). (**B**) 10% BRAF^V600E^ cocultured monolayers were seeded as described in methods. After 24 hr with doxycycline (2 µg/ml), monolayers were imaged by spinning disk confocal. Representative orthogonal Z projections and probability densities for nuclear height of inducible (green) and neighboring (grey) cells are shown (see methods). Extrusion (ΔZ) is calculated as the height difference between gaussian-fitted maxima of the green and black distributions. (**C**) 10% cocultures of indicated parental or inducible cells were treated with 24 hr doxycycline (2 μg/ml), imaged, and analyzed as in B. Data represents difference in nuclear height (ΔZ) for n = 18 observations normalized to the mean height of parental cells (dashed line), with mean and +/- standard deviation (black bars). Significance was calculated by two-sample t-test with ‘ns’ indicating no significance, ***p<0.001. (**D**) Representative basal and apical images (+6 µm) of WT or ADAM17^KO^, BRAF^V600E^ inducible cells (green) in WT monolayers (red) after 24 hr of doxycycline treatment. (**E**) 10% BRAF^V600E^ cocultures were pretreated with inhibitors (MEKi, 5 μM PD0325901, MPi, 5 μM Batimastat, EGFRi, 5 μM Gefitinib) and 24 hr doxycycline (2 μg/ml) or media, imaged and analyzed as in B. Data represents difference in nuclear height (ΔZ) for n ≥﻿ 16 independent observations presented as in C. (**F**) Inducible MEK2^DD^ cells were plated in 1% cocultures and treated with doxycycline (2 µg/ml) in the presence of MEK inhibitor (MEKi, 5 µM PD0325901) or amphiregulin function-blocking antibody (AREG FB Ab, 50 ng/ml) as indicated. Radial histograms are presented as in A for angles of n > 100 cells from two to three independent observations per condition. Data was assessed using subsampling and a two-sample KS test with ‘ns’ not significant, ***p<0.001 (see methods). (**G**) 10% MEK2^DD^ cocultures were pretreated with MEK inhibitor (MEKi, 5 µM PD0325901) or Amphiregulin function-blocking antibody (AREG FB Ab, 50 ng/ml) and 24 hr doxycycline (2 µg/ml) or media, as indicated, then imaged and analyzed as in B-C. Data represents difference in nuclear height (ΔZ) for n ≥ 11 independent observations normalized to the mean height of media-treated MEK2^DD^ cells (dashed line), with mean and +/- standard deviation (black bars). Significance was calculated by two-sample t-test with ‘ns’ indicating no significance, **p<0.01, and ***p<0.001.

In addition to proliferation, ERK waves have been shown to orient collective cell migration during wound healing (Aoki et al., 2017). In cocultures, sustained ERK activity in B-Raf^V600E^-inducible cells correlated with neighboring cell migration towards inducible cells in an ADAM17 and EGFR-dependent manner (Figure 5A). We hypothesized that coordinated migration of neighboring cells could physically contribute to oncogenic cell extrusion (Hogan et al., 2009; Leung and Brugge, 2012; Slattum et al., 2014). To address this hypothesis, we used confocal Z stacks to quantify extrusion of oncogene-expressing cells from monolayers (Figure 5B and Figure 5—Video 1). Interestingly, while pulsatile ERK activity (i.e. EGFR and B-Raf) was not sufficient to extrude cells, sustained ERK activity (i.e. B-Raf^V600E^ and MEK2^DD^) led to efficient epithelial cell extrusion apically (Figure 5C). KRAS^G12V^ induction did not result in apical extrusion to the extent observed for HRAS^G12V ^(Hogan et al., 2009, Figure 5—figure supplement 1). However, since sustained ERK activation in KRAS^G12V^ occurs later than B-Raf^V600E^ (Figure 1—figure supplement 1) further apical extrusion may also occur at a later time. Taken together, our data suggests that apical extrusion occurs when oncogenic perturbations trigger sustained ERK activity.

**Figure 5—figure supplement 1.**
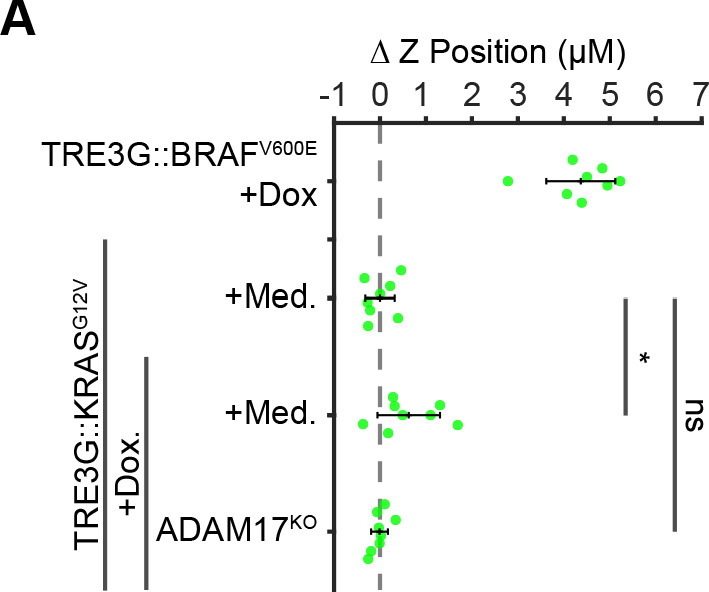
KRAS^G12V^ expressing cells do not extrude at 24 hr. (**A**) 10% BRAF^V600E^ or KRAS^G12V^ (WT or ADAM17^KO^) cocultures were treated with 24 hr doxycycline (2 µg/ml) or media, imaged and analyzed as in Figure 5B. Data represents difference in nuclear height (ΔZ) for n = 8 independent observations normalized to the mean height of un-induced KRAS^G12V^ cells (dashed line), with mean and +/- standard deviation (black bars). Significance was calculated by two-sample t-test with ‘ns’ indicating no significance, *p<0.05.

**Figure 5—figure supplement 2.**
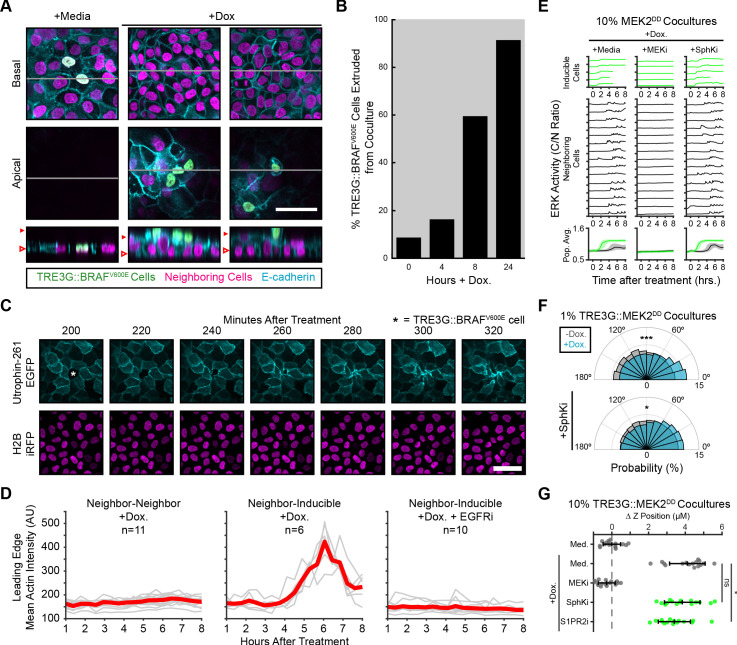
Actin dynamics and sphingosine kinase requirements during oncogenic cell extrusion. (**A**) Representative images of immunofluorescence for E-cadherin (cyan) from 1% BRAF^V600E^ cocultures treated for 24 hr with doxycycline (2 µg/ml) or media imaged by confocal. TRE3G::BRAF^V600E^ cells carry H2B-mClover nuclear markers (green), while neighboring cells have only H2B-iRFP (magenta). A basal and an apical plane are shown for each position, while the bottom shows an XZ orthogonal slice from the indicated position (grey line) in the above images. Orthogonal view shows positions of basal (hollow triangle) and apical planes (solid triangle). Scale bar = 50 µm. (**B**) Manual quantification of fully extruded TRE3G::BRAF^V600E^ cells from cocultures induced for indicated times (0, 4, 8, 24 hr) with doxycycline (2 µg/ml) after membrane immunofluorescence staining of E-Cadherin. In order to be considered fully extruded rather than elongated, cells must have no evidence of basal attachment. Data represents n ≥ 35 cells. (**C**) Time-course of actin dynamics and basal protrusions of neighboring cells in 1% BRAF^V600E^ coculture imaged with Utrophin-261-EGFP (cyan, top, see Figure 5—Video 2). TRE3G::BRAF^V600E^ cell is indicated by bold asterisk (*), and H2B-iRFP (bottom, magenta) from cells in the plane of the monolayer are shown to observe timing of extrusion. Scale bar = 50 µm. (**D**) Quantification of actin enrichment from BRAF^V600E^ cocultures. Median actin intensities (AUs) from randomly selected neighbor-neighbor cell borders are compared to the leading edges of all neighboring cells adjacent to TRE3G::BRAF^V600E^ cells that were treated with doxycycline alone (2 µg/ml, from images in C) or with doxycycline plus EGFR inhibitor (Gefitinib, 5 µM). See Materials and methods for details. (**E**) MEK2^DD^ cocultures were plated as in 2B, and treated with doxycycline (+Dox, 2 µg/ml) in the presence of media or inhibitors (MEKi, 5 µM PD0325901; SphKi, 10 µM SKII). Single-cell ERK activity traces are presented as in 2C with population averages and 25^th^-75^th^ percentiles (shaded) shown for n > 1000 cells per coculture condition. (**F**) Neighboring cell migration angles from 1% Inducible MEK2^DD^ cells cocultures treated with doxycycline (2 µg/ml) in the presence or absence of Sphingosine Kinase inhibitor (5 µM SKII). Data represents angles from n > 100 cells from two independent observations per condition presented as in 5A, and Dox condition reproduced from Figure 5F for comparison. Data was assessed using subsampling and a two-sample KS test with *p<0.05, ***p<0.001 (see Materials and mmethods). (**G**) Extrusion from 10% MEK2^DD^ cocultures pretreated with Sphinosine-1-Phosphate pathway inhibitors (SphKi, 10 µM SKII; S1PR2i, 10 µM JTE-013) and 24 hr doxycycline (2 µg/ml) or media, imaged and analyzed as in 5B-D (green). Some conditions reproduced from Figure 5 for comparison (grey). Data represents difference in nuclear height (ΔZ) for n ≥ 15 independent observations normalized to the mean height of parental cells (dashed line), with mean and +/- standard deviation (black bars). Significance was calculated by two-sample t-test with ‘ns’ indicating no significance, *p<0.05.

**Figure 5—video 1.** Extrusion of BRAF^V600E^-Expressing Cells. Cocultured monolayers of inducible BRAF^V600E^ cells were imaged every 10 min for 24 hr following doxycycline (2 µg/ml) treatment. The first frame shows the starting location of inducible cells (H2B-mClover, green) and neighboring cells (H2B-iRFP, magenta). The following video indicates cells in a basal position within the monolayer in magenta, and cells in an apical z-plane (+8 µm, cyan). Scale bar = 100 µm.

**Figure 5—video 2.** Live actin dynamics during extrusion. Inducible BRAF^V600E^ cells were cocultured with neighboring cells stably expressing Utrophin-261-EGFP, an actin-binding protein (cyan, see Figure 5—figure supplement 2). Actin dynamics and basal protrusions of neighboring cells (cyan) and the nuclei of all cells (H2B-iRFP, magenta), were imaged within the basal plane of the monolayer every 20 min from hours 1–7 after addition of doxycycline (2 µg/ml). Scale Bar = 50 µm.

In mammalian epithelia, apical extrusion eliminates apoptotic cells or crowded cells to maintain homeostasis (Rosenblatt et al., 2001; Eisenhoffer et al., 2012). Similar, but mechanistically different, apical extrusion has been observed for some oncogenic cells during EDAC (Kajita and Fujita, 2015). We wanted to know whether the extrusion of inducible cells with sustained ERK activity resembled EDAC. To differentiate between pseudostratified or de-laminated (Grieve and Rabouille, 2014) epithelium and extrusion we analyzed confocal images of E-cad membrane staining in induced cocultures. Cells with sustained ERK activity were fully extruded, sitting above WT cells in the plane of the monolayer (Figure 5—figure supplement 2). These images also demonstrate maintenance of E-Cad at the junctions between WT cells below extruded cells. Quantification of fully-extruded cells at several timepoints showed the majority of oncogenic cells being extruded from 4 to 8 hr after induction, but continuing until 24 hr, when 91% of inducible cells are fully extruded.

Both the oncogenic and apoptotic extrusion models involve cytoskeletal rearrangements at the site of extrusion (Rosenblatt et al., 2001; Kajita and Fujita, 2015). To observe live actin dynamics in cocultures, we made cell lines stably expressing Utrophin-261-EGFP (Belin et al., 2014). Using this tool, we observed transient accumulation of actin at the basal interface of B-Raf^V600E^ expressing and neighboring cells that first closed off the basal attachments of inducible cells before they were pushed apically out of the monolayer (Figure 5—figure supplement 2 and Figure 5—Video 2). These polarized, actin-containing basal protrusions were dependent on EGFR activity as they could be inhibited by EGFR inhibitor. Apoptotic extrusion relies on Sphingosine-1-phosphate (S1P) signaling through intrinsic S1P production and juxtracrine activation of the GPCR S1PR2 (Gu et al., 2011), yet inhibition of S1P production had only moderate effects on apical extrusion of MEK2^DD^ cells (Figure 5—figure supplement 2). Together, our results suggest that the apical extrusion of oncogenic cells observed in our experiments are similar to the EDAC mechanism previously described for HRAS^G12V^, V-Src, and other cells (Hogan et al., 2009; Kajita et al., 2010; Kajita and Fujita, 2015).

The requirement for paracrine signals in collective migration led to the question of whether paracrine signals were also required for extrusion. To test the role of ADAM17-mediated AREG-EGFR paracrine signals in promoting extrusion, we performed extrusion assays using ADAM17^KO^ cells or in the presence of EGFR inhibitor or AREG function-blocking antibodies. Extrusion of inducible cells was abolished in these conditions (Figure 5D–G), suggesting that ERK signaling waves are required for extrusion. Of note, since ADAM17^KO^ and EGFR inhibition affect ERK activation of neighboring cells without altering ERK dynamics in inducible cells, we hypothesized that that activation of inducible cells alone is not sufficient for extrusion, but that neighboring cell ERK activation may be required. To address this question, ERK-independent ADAM17 activation is needed.

**Figure 6.**
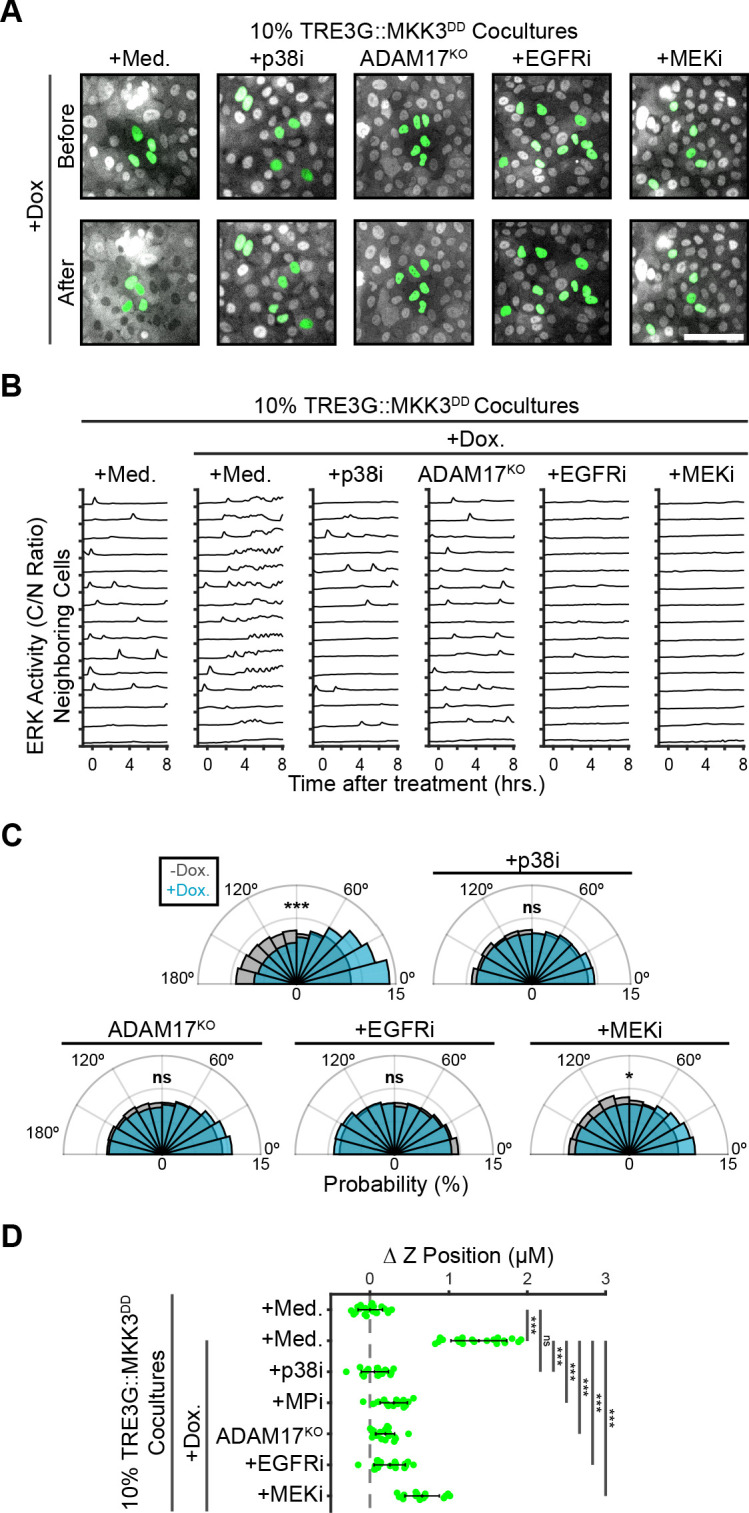
ERK activity in neighboring cells is required for coordinating extrusion. (**A**) Representative images showing WT or ADAM17^KO^ cells with inducible MKK3^DD^ (green), cocultured at 10% with neighboring ERK-KTR cells (grey). Cocultures were treated with doxycycline (2 µg/m) in the presence of media, p38 inhibitor (5 µM BIRB-796), EGFR inhibitor (5 µM Gefitinib), or MEK inhibitor (5 µM PD 0325901). Scale bar = 100 µm. (**B**) ERK activity traces of neighboring cells in coculture with MKK3^DD^-inducible cells (WT or ADAM17^KO^) plated at 10%, pretreated with inhibitors (p38i, 5 µM BIRB 796; EGFRi, 5 µM Gefitinib; MEKi, 5 µM PD 0325901) and doxycycline (2 µg/ml) or media, and imaged as in Figure 2C. 15 representative neighboring cell ERK activity traces are shown for each condition. (**C**) Inducible MKK3^DD^ cells (WT or ADAM17^KO^) were plated in 1% cocultures and treated with doxycycline (2 µg/ml) in the presence or absence of inhibitors. Radial histograms of migration angles before (grey) and 6–9 hr after (cyan) induction presented as in Figure 5A. Data represents angles from n > 900 cells from ≥6 observations per condition assessed using subsampling and a two-sample KS test with ‘ns’ not significant, *p<0.05, ***p<0.001 (see Materials and methods). (**D**) 10% MKK3^DD^ cocultures were pretreated with inhibitors (p38i, 5 µM BIRB 796; EGFRi, 5 µM Gefitinib; MEKi, 5 µM PD 0325901) and 24 hr doxycycline (2 µg/ml) or media, imaged and analyzed as in Figure 5B–D. Data represents difference in nuclear height (ΔZ) for n ≥ 16 observations normalized to the mean height of parental cells (dashed line), with mean and +/- standard deviation (black bars). Significance was calculated by two-sample t-test with ‘ns’ indicating no significance, ***p<0.001.

Previous studies have shown that the stress MAPK p38 phosphorylates and activates ADAM17 (Xu and Derynck, 2010). Thus, we used our doxycycline-inducible system to drive the expression of MKK3^DD^, a constitutively-active MAP2K specific for p38 (Figure 6—figure supplement 1), to activate ADAM17 independently of ERK. As expected, we found that p38 activation leads to ERK signaling waves (Figure 6A–B), proliferation (Xu and Derynck, 2010; Figure 6—figure supplement 2), directed migration (Figure 6C) and extrusion in an ADAM17 and EGFR dependent manner (Figure 6D and Figure 6—Video 1). However, B-Raf^V600E^ oncogenic signaling, extrusion and proliferation were unaffected by p38 inhibition (Figure 6—figure supplement 3), suggesting that sustained ERK or p38 activity are each capable of activating ADAM17 paracrine signaling. Using this ERK-independent MKK3^DD^ extrusion system, we found that MEK inhibition decreased directed migration and prevented extrusion, confirming that extrusion requires ERK activity in the neighboring cells (Figure 6C–D). Thus, ERK activity is required for extrusion in both oncogenic and neighboring cells with qualitatively different temporal dynamics. Notably, inhibition of S1P signaling in MKK3^DD^ cocultures also reduced extrusion efficiency despite having unaffected signaling (Figure 6—figure supplement 4). This result suggests that cell-autonomous ERK or p38 activation in extruded cells may underlie the fundamental differences reported between extrusion of oncogenic and stressed cells.

**Figure 6—figure supplement 1.**
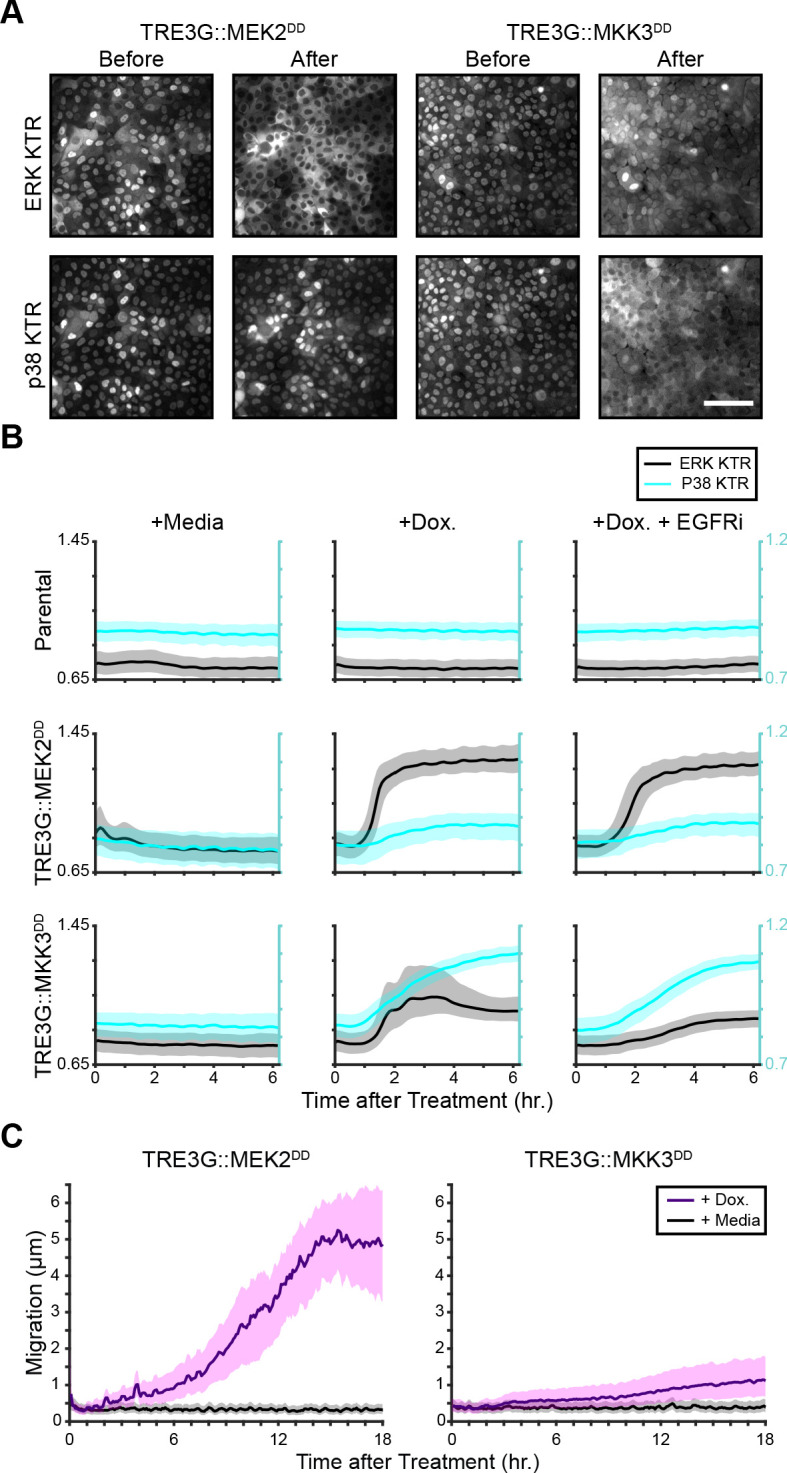
MAPK specificity of MEK2^DD^ and MKK3^DD^. (**A**) Representative images from inducible MEK2DD and MKK3DD cells expressing H2B-iRFP, ERK KTR-mCer3 and p38 KTR-mClov before and after induction with doxycycline (2 µg/ml). Scale bar = 100 µm. (**B**) Population average traces of ERK (black) and p38 (cyan) activity for indicated parental and inducible ERK/p38 KTR cells from A. Cocultures were treated with media, doxycycline, or doxycycline with EGFR inhibitor (5 µM gefitinib) to limit paracrine ERK activation. Population averages and 25^th^-75^th^ percentiles (shaded regions) are shown for n > 2000 cells. (**C**) Migration of inducible cells from B plotted as µm change for every 5 min timepoint.

**Figure 6—figure supplement 2.**
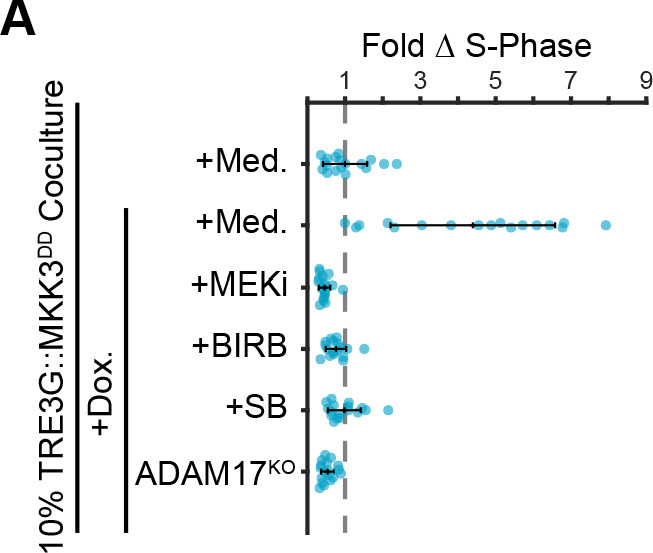
Mosaic p38 activation leads to ADAM17-EGFR -ependent proliferation of neighboring cells. (**A**) EdU of 10% MKK3^DD^ inducible cell cocultures treated for 24 hr with vehicle (+Media) or doxycycline (2 µg/ml) in the presence of MEK inhibitor (MEKi, 5 µM PD 0325901), p38 inhibitiors (5 µM BIRB 796 or 25 µM SB 203580) or in ADAM17^KO^ cells. Data presented as in 4B for n ≥ 16 observations. See Materials and methods for details about EdU incorporation.

**Figure 6—figure supplement 3.**
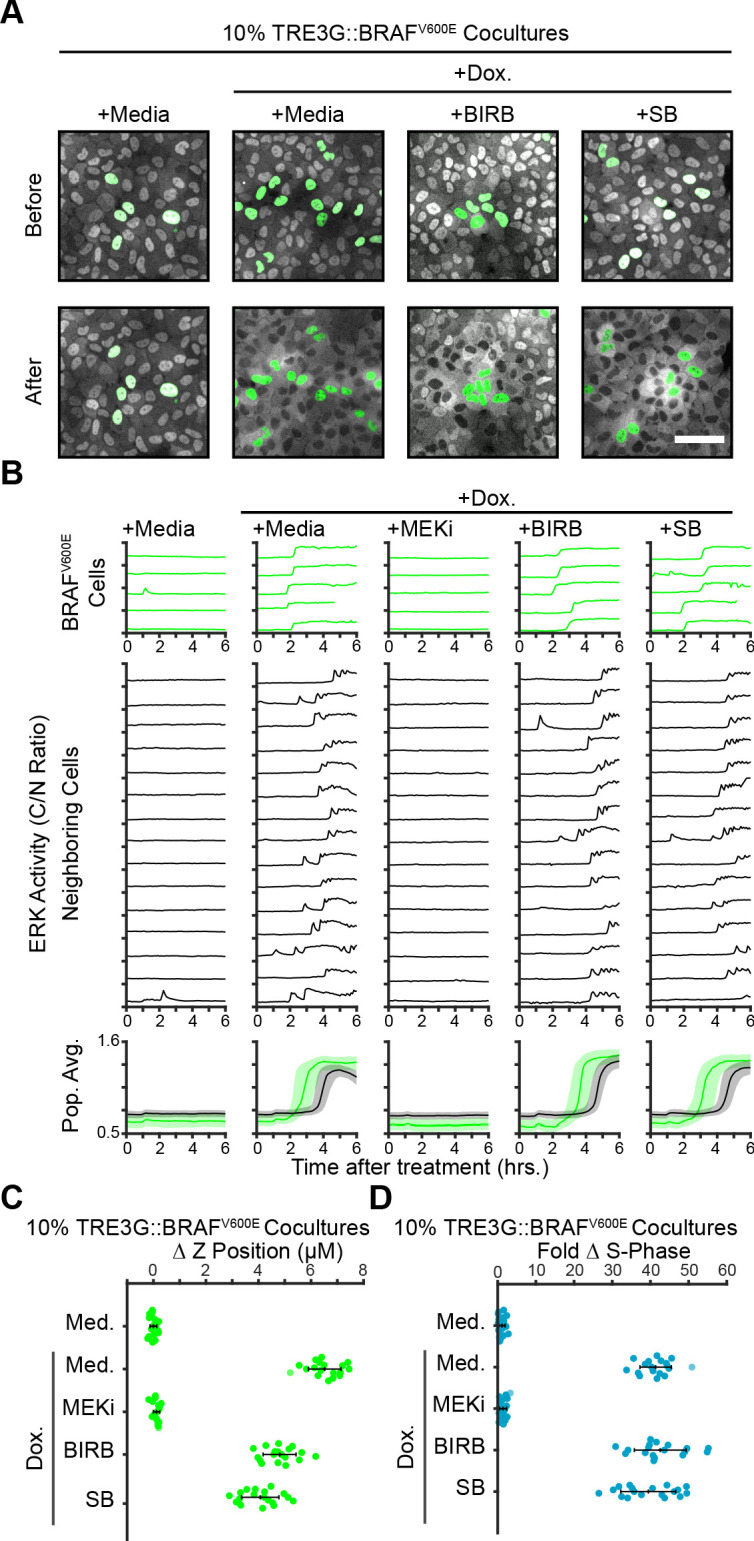
Oncogene-induced paracrine ERK activity and resulting cell behaviors are p38-independent. (**A**) Representative images from 10% TRE3G::BRAF^V600E^ cocultures plated as in 2B before and after treatment with media or doxycycline (2 µg/ml) alone or with p38 inhibitors (5 µM BIRB 796 or 25 µM SB 203580). Scale bar = 100 µm. (**B**) ERK activity traces, averages and 25^th^-75^th^ percentiles from inducible BRAF^V600E^ cocultures from a represented as in 2C for n > 1600 cells. (**C**) Extrusion of BRAF^V600E^ inducible cells from 10% cocultures treated as indicated and presented as in 5C for n = 18 observations. (**D**) EdU incorporation in 10% BRAF^V600E^ cocultures treated as indicated and presented as in Figure 4B for n = 18 observations (see Materials and methods).

**Figure 6—figure supplement 4.**
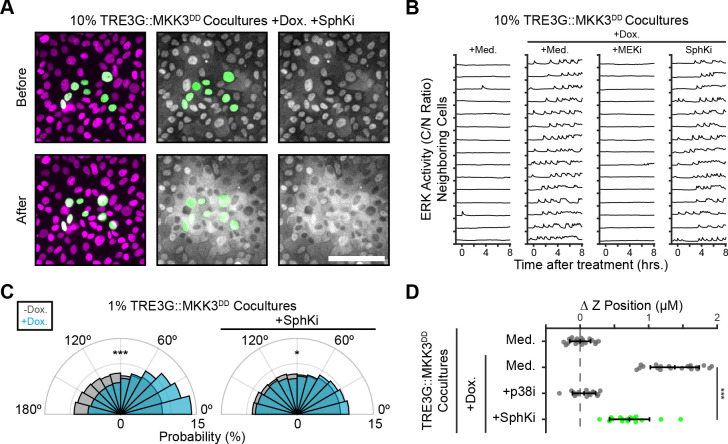
Partial involvement of S1P signaling in extrusion of p38-active cells. (**A**) Representative images from MKK3^DD^ cocultures pretreated with sphingosine kinase inhibitor (SphKi, 10 µM SKII) before induction with doxycycline (2 µg/ml). Scale bar = 100 µm. (**B**) ERK activity traces of neighboring cells in coculture with MKK3^DD^ inducible cells plated at 10%, pretreated with inhibitors (MEKi, 5 µM PD 0325901; SphKi, 10 µM SKII) and doxycycline (2 µg/ml) or media, imaged as in 2C and presented as in 6B. 15 representative neighboring cell ERK activity traces are shown for each condition. (**C**) Inducible MKK3^DD^ cells were plated in 1% cocultures and treated with doxycycline (2 µg/ml) in the presence of Sphingosine Kinase inhibitor (10 µM SKII). Radial histograms of migration angles before (grey) and after (cyan) induction presented as in Figure 6C, and the +Dox alone condition (left) is reproduced from Figure 6C for comparison. Data represents angles from n > 900 cells from ≥6 observations per condition assessed using subsampling and a two-sample KS test with *p<0.05, ***p<0.001 (see methods). (**D**) 10% MKK3^DD^ cocultures were pretreated with inhibitors (p38i, 5 µM BIRB 796; SphKi, 10 µM SKII) and 24 hr doxycycline (2 µg/ml) or media, imaged and analyzed as in 5B-D. Data represents difference in nuclear height (ΔZ) for n ≥ 16 observations normalized to the mean height of parental cells (dashed line), with mean and +/- standard deviation (black bars). Some conditions are reproduced from Figure 6D for comparison (grey). Significance was calculated by two-sample t-test with ***p<0.001.

**Figure 6—video 1.** p38-induced ERK signaling waves. Inducible MKK3^DD^ cells with a H2B-mClover nuclear marker (green) were cocultured in monolayers with neighboring cells (H2B-iRFP, magenta) (see Figure 4). Images were collected every 5 min for an hour basal period and 24 hr following addition of doxycycline (2 µg/ml) at time 0. ERK KTR-mCer3 (grey) reports ERK activity in all cells. Three examples are shown for both WT (left) and ADAM17^KO^ (right) inducible cells. Scale bar = 100 µm.

**Figure 7.**
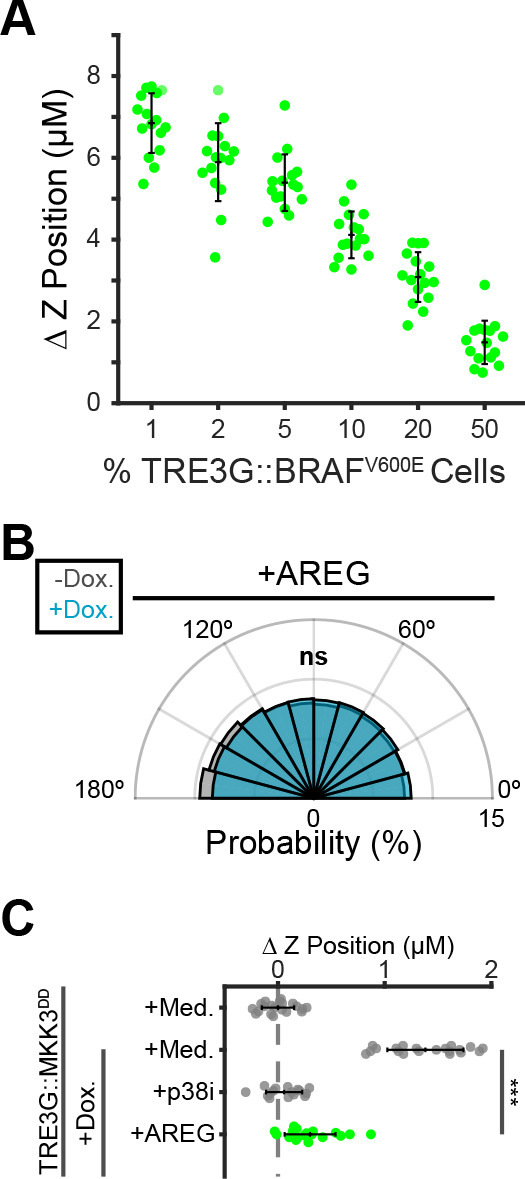
Localized paracrine signals coordinate directed migration and extrusion. (**A**) Inverse relationship between fraction of oncogenic cells in coculture and extrusion efficiency. Inducible BRAF^V600E^ cells were cocultured at indicated proportions, treated 24 hr with doxycycline (2 µg/ml), imaged, and analyzed as in Figure 5B. Data represents difference in nuclear height (ΔZ) for n ≥ 15 observations presented as in 5C. (**B**) Inducible MKK3^DD^ cells were plated in 1% cocultures and treated with doxycycline (2 µg/ml) in the presence or absence of Amphiregulin (20 ng/ml). Radial histograms of migration angles before (grey) and after (cyan) induction presented as in Figure 6C. Data represents angles of n > 900 cells from ≥6 observations assessed using subsampling and a two-sample KS test with ‘ns’ not significant, ***p<0.001 (see Materials and methods). (**C**) 10% MKK3^DD^ cocultures were pretreated with Amphiregulin (20 ng/ml, green) and 24 hr doxycycline (2 µg/ml) or media, imaged and analyzed as in Figure 5B–D, and compared to selected conditions reproduced from Figure 6D (grey). Data represents difference in nuclear height (ΔZ) for n ≥ 16 observations normalized to the mean height of parental cells (dashed line), with mean and +/- standard deviation (black bars). Significance was calculated by two-sample t-test with ‘ns’ indicating no significance, ***p<0.001.

**Figure 8.**
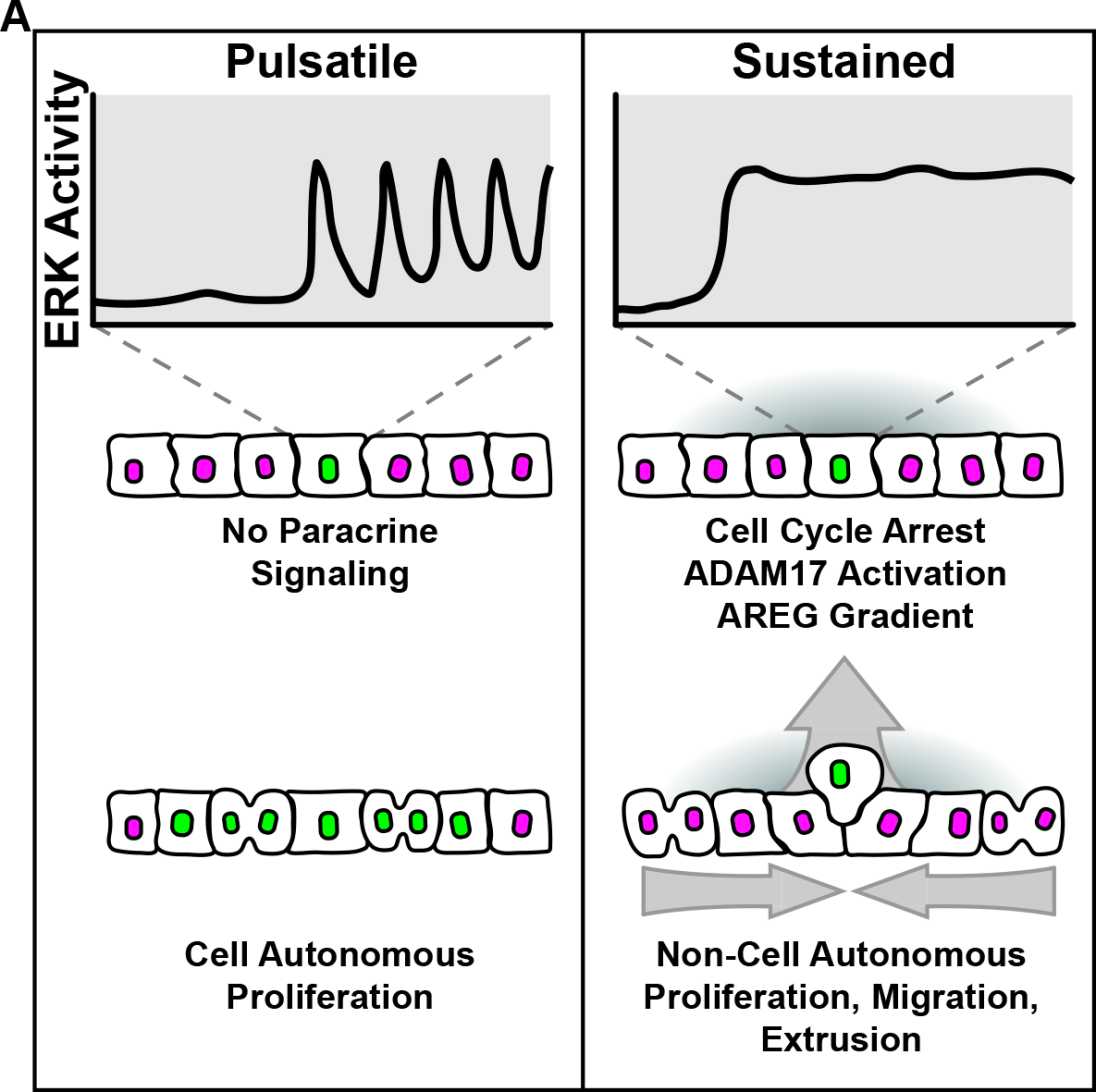
Graphical summary. (**A**) Model summarizing cell autonomous and non-cell autonomous effects resulting from pulsatile and sustained ERK signaling dynamics.

Finally, we asked whether the spatiotemporal properties of paracrine ERK signaling waves are important to coordinate extrusion. We first tested the efficiency of extrusion with altered proportions of B-Raf^V600E^ cells in the coculture, as higher proportions will have de-centralized and overlapping signaling events. The proportion of inducible cells was inversely correlated with extrusion efficiency (Figure 7A). Moreover, exogenous addition of AREG, which triggers widespread ERK activation preventing any spatially defined waves, eliminated directed migration of neighboring cells and extrusion (Figure 7B–C). The observation that in cocultures, polarized actin enrichment in neighboring cell basal protrusions is absent with EGFR inhibition, also indicates that growth factor signaling provides directional information during extrusion (Figure 5—figure supplement 2). Together this data suggests that locally generated paracrine signaling coordinates directed migration of neighboring epithelia to promote extrusion of oncogenic cells (Figure 8).

## Discussion

A wide variety of ERK pathway alterations occur across human tumors, often resulting in different cancer phenotypes (Sanchez-Vega et al., 2018; Bailey et al., 2018; Hoadley et al., 2018). To better understand the signaling effects of oncogenic mutations, we used live cell imaging of signaling biosensors upon induction of oncogenes and found that different ERK pathway oncogenes trigger two distinct temporal patterns: pulsatile or sustained ERK activity. While our approach is admittedly different than acquisition of point mutations in vivo, ERK dynamics resulting from oncogene overexpression robustly correlated with the same cellular phenotypes: (i) pulsatile ERK activity correlates with increased proliferation and, (ii) sustained ERK activity leads to cell cycle arrest similar to OIS (Hahn and Weinberg, 2002; Courtois-Cox et al., 2006; Kuilman et al., 2008). Moreover, we showed that sustained ERK activity in oncogenic cells triggers ERK signaling waves through unperturbed neighboring cells. These signaling waves depend on the ADAM17-EGFR paracrine signaling axis and lead to different non cell-autonomous behaviors such as (i) proliferation, (ii) directed migration of neighboring cells toward oncogenic cells, and (iii) oncogenic cell extrusion (Figure 8).

Our data indicates that cancer mutations can have non-cell autonomous contributions to tissue growth (Figure 4). Interestingly, studies in mouse epidermis have shown that mosaic oncogene expression promotes proliferation of wild type surrounding cells, which is required to expel mutant outgrowths from the tissue (Brown et al., 2017). While the role of ADAM17 in this phenomenon and during early tumorigenesis in vivo is yet unknown, it is tempting to speculate that polypous outgrowths may occur in the presence of non-proliferative oncogenic cells that release growth factors via ADAM17. The mechanisms that mediate tissue expelling in vivo remain unknown.

Previous work in described the process of oncogenic cell extrusion as part of the so called Epithelial Defense Against Cancer (i.e. EDAC) (Hogan et al., 2009). However, the signals involved in recognition of oncogenic cells, and why only specific oncogenes trigger oncogenic cell extrusion was unclear (Kajita and Fujita, 2015; Clavería and Torres, 2016; Maruyama and Fujita, 2017). Our data suggests that perturbations that elicit sustained ERK activity (eg. B-Raf^V600E^, MEK2^DD^), activate ADAM17, which in turn releases EGFR ligands (Figure 3). This paracrine signal is critical for oncogenic extrusion (Figure 5). However, we acknowledge that different cellular states such as apoptosis or overcrowding lead to extrusion by different mechanisms. Of note, our data showed that ERK activation drives extrusion to a higher extent than p38 activation (Figure 6), which may result from a difference in overall cell autonomous migration in these two cases (Figure 6—figure supplement 1). Moreover, sphingosine kinase inhibition caused greater defects in extrusion of p38-active cells than ERK-active cells (Figure 5—figure supplement 2 and Figure 6—figure supplement 4). This finding agrees with work showing that EDAC of transformed HRAS^G12V^ cells is less dependent on sphingosine-1-phosphate production than extrusion of crowded or apoptotic cells (Yamamoto et al., 2016), where stress signaling may be involved.

We and others have identified AREG as one of the key EGFR ligands in mammary epithelial cells (Sternlicht et al., 2005; Figure 3); however, different ligands may be required in other tissues. These ligands, released by ADAM17, coordinate the migration of neighboring cells by mechanisms that remain unclear. Cultured monolayers are fundamentally different than in vivo tissues; however, the chemo-attractive properties of growth factors for directed migration have been modeled and studied in cell culture (Devreotes et al., 2017; Tranquillo et al., 1988). We propose that local signaling gradients are created by oncogenic cells to coordinate directed migration of neighbors. To support this idea, we show that addition of exogenous AREG or increased fractions of oncogenic cells both prevent directed migration of neighboring cells (Figure 7), and that during extrusion, polarized actin-containing basal protrusions require growth factor signaling (Figure 5—figure supplement 2). Localized ERK signaling gradients have also been observed during morphogenesis of *Drosophila*, avian, and mammalian embryos (Yang et al., 2002; Ogura et al., 2018; Corson et al., 2003), and in preserving homeostasis of mammalian epidermis and intestinal organoids (Hiratsuka et al., 2015; Muta et al., 2018; Liang et al., 2017). Thus, in addition to roles in oncogenesis, the ADAM17-EGFR paracrine signaling axis may direct collective behaviors during development.

Overall, our results highlight the importance of quantitative live-cell approaches to understand the effects of genetic perturbations and cell-cell communication in tissues. We propose a critical role for ERK signaling dynamics and the ADAM17-EGFR signaling axis in coordinating cell behaviors at the tissue level.

## Materials and methods

### Cell lines & reagents

 MCF10A human mammary epithelial cells (ATCC) were grown at 37° and 5% CO_2_ in DMEM/F12 (Gibco) with 5% horse serum (HS) (Sigma), 10 µg/ml Insulin (Sigma), 20 ng/ml EGF (Peprotech), 1x Penicillin-Streptomycin (P/S) (Gibco), 0.5 mg/ml Hydrocortisone (Sigma), 100 ng/ml Cholera Toxin (Sigma). Cells were passaged every 3 days with 0.25% Trypsin-EDTA (Gibco), are mycoplasma free, and were verified by STR-profiling (ATCC).

Cell lines were generated with lentivirus produced in HEK293-FTs (Thermo) with third-generation packaging plasmids and Lipofectamine 2000 (Thermo). Viral supernatants were collected 48 hr after transfection and incubated in MCF10As with polybrene (10 µg/ml, EMD Millipore). To create dual-sensor cells, MCF10As were infected with a lentiviral H2B-iRFP vector (Addgene) and sorted. We used gateway cloning (Campeau et al., 2009) to introduce ERK-KTR-mCer3 and ERK1-mRuby2 into PGK pLenti DEST vectors (Addgene), infected and selected the H2B-iRFP MCF10As (Blasticidin 3 µg/ml and Hygromycin 10 µg/ml Corning). We isolated moderately expressing clones using cloning cylinders (EMD Milipore). For inducible cells, a gateway-ready reverse TET trans-activator (rtTA) plasmid was created by adding the rtTA with a 2A peptide to the Puromycin resistance gene in a CMV Puro DEST plasmid (Addgene) by gibson cloning (Gibson et al., 2009). Human coding sequences were acquired from either Addgene or the Thermo Ultimate ORF Collection, sequence verified, and introduced in the rtTA CMV Puro DEST plasmid by gateway cloning (Campeau et al., 2009). These plasmids were used for lentivirus, and infected cells were selected with Puromycin (1 µg/ml, Sigma). Utrophin-261-EGFP cell lines were made by cloning the coding region from pEGFP-C1 Utr261-EGFP (Addgene) into a pENTR backbone by Gibson cloning, and then introduced into the pLenti PGK Puro DEST plasmid by gateway cloning. These plasmids were used to generate lentivirus, and infected cells were selected with Puromycin.

For inhibitor experiments, small molecules or antibodies and doxycycline were dissolved to a 10X working concentration in imaging media before addition. Final DMSO concentration did not exceed 0.15%. Inhibitors used include the MEK inhibitor PD-0325901, the MMP/ADAM inhibitor Batimastat, the EGFR inhibitor Gefitinib, the p38 inhibitor BIRB-796, the Sphingosine Kinase inhibitor SKII, and the S1PR2, inhibitor JTE-013 all from Selleck Chemicals. The p38 inhibitor SB-203580 was obtained from Sigma. Amphiregulin was ordered from Peprotech. Amphiregulin function-blocking antibody is from R and D systems.

The ADAM17^KO^ cell lines were created using the CRISPR V2 Neo system (a gift from Dr. Andrew Holland) and gRNA oligos targeting R241 of exon 6. Dual sensor cells were infected with lentivirus carrying this plasmid, selected with Neomycin (500 µg/ml, Sigma) and clonally expanded before western blot validation (Figure 2B).

### Live imaging

Cells were plated at 3*10^5^ cells/well in fibronectin-treated (EMD Millipore) 96-well glass-bottom plates (Thermo Scientific) 48 hr before imaging. The following day, monolayers were serum-starved with 0.5% HS, phenol-red-free DMEM/F12 containing P/S with 1 mM Na Pyruvate and 10 mM HEPES. For signaling experiments in Figure 1 and Figure 1—figure supplement 1, media was switched to 0% HS several hours before imaging to limit basal signaling. Monolayers were imaged using a Metamorph-controlled Nikon Eclipse Ti-E epifluorescence microscope with a 20x air objective and a Hamamatsu sCMOS camera. The multi LED light source SpectraX (Lumencor) and the multiband dichroic mirrors DAPI/FITC/Cy3/Cy5 and CFP/YPF/mCherry (Chroma) where used for illumination and imaging without any spectral overlap. For extrusion and live-actin experiments, a Metamorph-controlled Nikon Eclipse Ti-E spinning-disc confocal (Yokogawa W1) with a 20x or 40X objective, Prime 95-B sCMOS camera (Photometrics) and a Multiline laser launch (Cairn Research) was used to capture H2B-iRFP and H2B-mClover or Utrophin-261-EGFP images every 1 µm of a 25–30 µm range through monolayers. Temperature (37°C), humidity and CO_2_ (5%) were maintained throughout all imaging using OKO Labs control units. Sample sizes were selected by attempting to capture at least 100 cells from each population, with several hundred cells preferred. Key conditions from imaging experiments were performed at least twice, with one replicate presented in figures.

### Image analysis and quantification

Primary time-lapse images were subjected to flat-fielding and registration (custom software Aikin et al., 2020) before object segmentation and measurements in Cell Profiler. Nuclear positions were used to track individual cells through time-series (custom software Aikin et al., 2020) and intensity ratios were calculated as previously described (Regot et al., 2014). Minimal cleaning of traces excluded cells where tracks switched between two objects, where the KTR ratios were affected by segmentation errors, or where traces represent less than two thirds of the entire time-course. In conditions where cells move rapidly, such as B-Raf^V600E^ and MEK2^DD^, and traces are shorter due tracking errors, track-length restraints were relaxed to include more cells for analysis. Single-cell traces were chosen by random plotting of distinct cells and selection of those that were tracked throughout the whole experiment. Peak counting was performed with software based on findPeaks (O'Haver, 2014; Mathworks.com) and modified to detect peaks based on the rate of change between gaussian-fitted minima and maxima from single-cell traces.

For directed migration, positions were selected where distinct groups of inducible cells were present in the center of the field of view. Migration was quantified by positional changes over 20 min intervals for specified time windows, from all WT neighboring cells within a 200 µM X 200 µM area centered on the group of inducible cells. The migration angles of neighboring cells are plotted as radial histograms where 0° indicates migration directly towards, and 180° directly away from the center of isolated inducible cell groups. Migration datasets contain many sampled angles from large populations of cells. To overcome issues with high power, we applied subsampling techniques using 1000 iterations of 1000 randomly-selected migration angles each, and presented the median Two-Sample Kolmogorov-Smirnov (KS) Test P-values from these iterations (‘ns’, not significant, *p<0.05, **p<0.01, ***p<0.001).

For extrusion experiments, histograms of mClover and mRuby pixel intensities across each z-stack were fit to gaussian curves using Matlab. The difference in gaussian fitted maxima of inducible cells and neighboring cells for each observation are plotted. Extrusion experiment sample size represents all non-overlapping positions from 2 to 3 independent wells excluding outliers resulting from imaging artifacts. Two-sample T-test significance values compare indicated conditions (‘ns’, not significant, *p<0.05, **p<0.01, ***p<0.001).

For live-actin imaging experiments, ERK-KTR, H2B-iRFP were infected with the TRE3G::BRAF^V600E^. These cells were plated in 1% coculture with neighboring ERK-KTR, H2B-iRFP cells containing the Utrophin-261-EGFP construct. Oncogenic cells were identified by lack of green fluorescence and confirmed by images of the KTR, showing activation after induction with doxycycline (2 µg/ml). Actin enrichment was quantified by manually tracing the border of adjacent Utrophin-261-EGFP cells at the leading edge in contact with oncogenic cells on Fiji. All cells from a single extrusion event are plotted along with their mean.

### Immunoblotting

For assessment of protein expression in Figure 1—figure supplement 2 , parental, TRE3G::BRAF^WT^ and TRE3G::BRAF^V600E^ cells were plated in 6-well plastic culture plates, and starved with 0.5% HS, DMEM/F12 containing P/S with 1 mM Na Pyruvate and 10 mM HEPES overnight before treatment with media or doxycycline (2 µg/ml) for 24 hr. Samples were lysed with RIPA buffer (CST) containing HALT protease and phosphatase inhibitors (Thermo), and reduced in Laemelli SDS buffer (BioRad) with BME (Sigma). Samples underwent electrophoresis on 4–15% gradient polyacrylamide gels (BioRad) and were immunoblotted with Rabbit anti-BRAF (CST) and mouse anti-HSC70 (Santa Cruz Biotechnology), and IRDye donkey anti-rabbit 800 and goat anti-mouse 680 secondary antibodies (LiCor) before imaging. For validation of ADAM17 CRISPR-KOs in Figure 3 , suspected clones were grown, lysed, and run on a gel as described above, before immunoblotting with Rabbit anti-ADAM17 (CST) and mouse anti-HSC70 (Santa Cruz Biotechnology) primary and IRDye donkey anti-rabbit 800 and goat anti-mouse 680 secondary antibodies (LiCor). All images were acquired on an Odyssey Infrared Scanner (LiCor).

### Proteomics

For mass spectrometry, cells were grown to 90% confluency in T175 flasks and serum starved 24 hr (see live imaging) before switching to 15 mL growth factor/serum-free DMEM/F12 +/- Dox for 4 hr. The supernatant was collected and concentrated using 3 kDa cut-off centrifugal filters (Millipore-Sigma). Triplicate samples were quantified by the Pierce Assay (Thermo Scientific), reduced, alkylated, and trypsin digested before labeling with Tandem Mass Tag labels. Peptide fractions were analyzed by LC/MSMS using an Easy-LC 1200 HPLC system interfaced with an Orbitrap Fusion Lumos Tribrid Mass Spectrometer (Thermo Fisher Scientific). Isotopically resolved masses in precursor and fragmentation spectra were processed in Proteome Discoverer software (v2.3, Thermo Scientific). Data were searched using Mascot (2.6.2, Matrix Science) against the 2017_Refseq 83 Human database and filtered at a 1% FDR confidence threshold.

### Cell proliferation assay

Monolayers were plated and starved as described above and treated with doxycycline (Dox, 2 µg/ml) in the presence of indicated inhibitors for 24 hr. During the final 4 hr, EdU (10 µM, Thermo Fischer Scientific) was added into cultures to label S phase cells then fixed with methanol and washed before Alexa-Fluor Azide 488 click labelling (Thermo Fischer Scientific) and DAPI staining (Thermo Scientific). Monolayers were imaged by epifluorescence. Because methanol fixation eliminates fluorescence from fluorescent proteins, cocultures were imaged just before fixation and registered with DAPI and EdU images to determine positions of inducible and neighboring cells. Sample size for population EdU experiments represents all non-overlapping positions from 2 to 3 independent wells, excluding outliers resulting from imaging artifacts. Key conditions were replicated at least twice.

### Immunofluorescence

Monolayers were plated and starved as described above, and treated with media or doxycycline (Dox, 2 µg/ml) in the presence of any indicated inhibitors for 24 hr or timepoints as marked. To induce EMT, parental cells were maintained in full serum supplemented with TGFβ (5 ng/ml or 50 ng/ml, R and D Systems) through splittings over 8 days to induce EMT (Hao et al., 2019), then cells were plated and starved as described with consistent TGFβ. Cells were fixed 15 min with 4% PFA in PBS, washed with PBS before incubating 1.5 hr in blocking buffer (PBS + 0.3% Triton X-100 + 5% BSA), followed by PBS washing and incubation overnight in blocking buffer with added primary antibodies (Rabbit anti-E-Cadherin, or Rabbit anti-N-Cadherin, both CST). The following day, cells were washed in PBS before incubating 2 hr in blocking buffer with secondary antibody (Donkey anti-Rabbit IgG Alexa Fluor 405, Abcam). Cells were then washed with PBS and stored at 4°C until imaging via spinning disk confocal as described above. All incubations occurred at room temp in the dark, except the overnight primary, which was incubated at 4°C.

## Data Availability

All data generated or analysed during this study are included in the manuscript and supporting files.

## Additional files

Supplementary file 1. Table of secreted factors identified by mass spectrometry. Supernatants from ADAM17^KO^ or WT cells expressing (+Dox) or not expressing (-Dox) BRAF^V600E^ were collected and analyzed by Tandem-Mass-Tag (TMT) mass spectrometry as described in methods. Table shows fold change and p-values for the 24 identified factors presented in Figure 3D in red.

Transparent reporting form

## Appendix 1

**Appendix 1—key resources table:**

Reagent type (species) or resource	Designation	Source or reference	Identifiers	Additional information
Cell line (Human)	MCF10A	ATCC		
Cell line (Human)	HEK293FT	Thermo-Fisher		
Recombinant DNA reagent	pLenti H2B-iRFP	PMID:24949979	H2B-iRFP; 'Nuclear marker'	pSR1881
Recombinant DNA reagent	pLenti PGK-ERK1-mRuby2	This paper	ERK Localization Sensor	pSR1214, Regot Lab
Recombinant DNA reagent	pLenti PGK-ERK-KTR-mCerulean3	Addgene #90229 PMID:24949979	ERK-KTR; ERK Kinase Translocation Reporter	pTA30, Regot Lab
Recombinant DNA reagent	pLenti H2B-mClover	This paper	H2B-Clover	pTA54, Regot Lab
Recombinant DNA reagent	pLenti PGK::rtTA, TRE3G::FGFR1^WT^	This paper	TRE3G::FGFR1^WT^	pTA46, Regot Lab
Recombinant DNA reagent	pLenti PGK::rtTA, TRE3G::FGFR2^WT^	This paper	TRE3G::FGFR2^WT^	pHC127, Regot Lab
Recombinant DNA reagent	pLenti PGK::rtTA, TRE3G::EGFR1^WT^	This paper	TRE3G::EGFR1^WT^	pHC132, Regot Lab
Recombinant DNA reagent	pLenti PGK::rtTA, TRE3G::HER2^WT^	This paper	TRE3G::HER2^WT^	pHC123, Regot Lab
Recombinant DNA reagent	pLenti PGK::rtTA, TRE3G::KRAS^WT^	This paper	TRE3G::KRAS^WT^	pHC131, Regot Lab
Recombinant DNA reagent	pLenti PGK::rtTA, TRE3G::KRAS^G12V^	This paper	TRE3G::KRAS^G12V^	pHC136, Regot Lab
Recombinant DNA reagent	pLenti PGK::rtTA, TRE3G::BRAF^WT^	This paper	TRE3G::BRAF^WT^	pHC142, Regot Lab
Recombinant DNA reagent	pLenti PGK::rtTA, TRE3G::BRAF^V600E^	This paper	TRE3G::BRAF^V600E^	pHC125, Regot Lab
Recombinant DNA reagent	pLenti PGK::rtTA, TRE3G::MEK1^WT^	This paper	TRE3G::MEK1^WT^	pHC134, Regot Lab
Recombinant DNA reagent	pLenti PGK::rtTA, TRE3G:: MEK1^DD^	This paper	TRE3G:: MEK1^DD^	pAP53, Regot Lab
Recombinant DNA reagent	pLenti PGK::rtTA, TRE3G:: MEK2^WT^	This paper	TRE3G:: MEK2^WT^	pHC126, Regot Lab
Recombinant DNA reagent	pLenti PGK::rtTA, TRE3G:: MEK2^DD^	This paper	TRE3G:: MEK2^DD^	pHC141, Regot Lab
Recombinant DNA reagent	pLenti PGK::rtTA, TRE3G:: MKK3^DD^	This paper	TRE3G:: MKK3^DD^	pAP55, Regot Lab
Recombinant DNA reagent	CMV Puro DEST	Addgene #17452 PMID:19657394		
Recombinant DNA reagent	PGK Puro DEST	Addgene #19068 PMID:19657394		
Recombinant DNA reagent	pEGFP-C1 Utr261-EGFP	Addgene #58471 PMID:26317264		
Recombinant DNA reagent	pLenti PGK-Utr261-EGFP puro	This paper		pTA152, Regot Lab
Recombinant DNA reagent	pLenti PGK-P38-KTR-mClover	This paper		pAP50, Regot Lab
Recombinant DNA reagent	pLenti PGK-JNK-KTR-mRuby2	Addgene #59154 PMID:24949979		pSR1846
recombinant DNA reagent	lentiCRISPR_V2_Neo	Gift from Dr. Andrew Holland		lentiCRISPR_V2_Puro on addgene as #52961
sequence-based reagent	ADAM17^KO^ guide	This paper, from IDT		5’-CTACAGATACATGGGCAGAG-3’ (targets R241 of exon 6)
Recombinant DNA reagent	pLenti CRISPR ADAM17^KO^ Neo	This paper	ADAM17^KO^	pTA70, Regot Lab
Chemical compound, drug	PD-0325901	Selleck Chemicals #S1036	MEKi; MEK inhibitor	5µM
Chemical compound, drug	Batimastat	Selleck Chemicals #S7155	MPi; MMP/ADAM inhibitor	5µM
Chemical compound, drug	Gefitinib	Selleck Chemicals #S1025	EGFRi; EGFR inhibitor	5µM
Chemical compound, drug	BIRB-796	Selleck Chemicals # S1574	P38i; p38 inhibitor; BIRB	5µM
Chemical compound, drug	SB-203580	Sigma # S8307	P38i; p38 inhibitor; SB	25µM
Chemical compound, drug	SKII	Selleck Chemicals #S7176	SKi; Sphingosine Kinase inhibitor	10µM
Chemical compound, drug	JTE-013	Selleck Chemicals # S128	S1PR2i; S1PR2 inhibitor	10µM
Peptide, recombinant protein	Doxycycline	Sigma #D9891	Dox	2μg/ml
Peptide, recombinant protein	Amphiregulin	Peprotech #100-55B	AREG	20ng/ml
Peptide, recombinant protein	TGFβ	R&D Systems #7754-BH	TGFβ	5ng/ml or 50ng/ml
Peptide, recombinant protein	EGF	Peprotech #AF-100-15	EGF	MCF10A culture
Peptide, recombinant protein	Insulin	Sigma #I0516		MCF10A culture
Peptide, recombinant protein	Penicillin/ Streptomycin	Sigma #P0781		MCF10A culture
Peptide, recombinant protein	Cholera Toxin	Sigma # C-8052		MCF10A culture
Peptide, recombinant protein	Hydrocortisone	Sigma #H-0888		MCF10A culture
Other	Horse Serum	Gibco #16050-122		MCF10A culture
Other	DMEM/F12	Gibco #11030-032		MCF10A culture
Other	0.25% Trypsin-EDTA	Gibco #25300-054		MCF10A culture
Other	Puromycin	Sigma #P8833		1 μg/ml
Other	Blasticidin	Corning #30-100-RB		3μg/ml
Other	Hygromycin	Gibco #10687010		10 μg/ml
Other	Neomycin	Sigma #N6386		500μg/ml
Other	Lipofectamine 2000	Thermo Fisher #11668-027		For lentiviral production
Other	Polybrene	EMD/Millipore #TR-1000-G		10 μg/ml, for lentiviral infection
Other	Human Plasma Fibronectin	EMD/Millipore #FC010		
Chemical compound, drug	EdU	Thermo Fisher # A10044	EdU	10μM
Chemical compound, drug	Alexa-Fluor Azide 488 click labelling	Thermo Fisher # A10266		
Chemical compound, drug	DAPI	Thermo Fisher # D3571		
Antibody	Anti-Amphiregulin Antibody (mouse monoclonal)	R & D Systems #MAB262	AREG FB-Ab	50ng/ml
Antibody	Anti-ADAM17 Antibody (rabbit polyclonal)	CST #3976S	α-ADAM17	1:1,000
Antibody	Anti-BRAF Antibody (rabbit monoclonal)	CST #14814S	α-BRAF	1:1,000
Antibody	Anti-E-Cadherin Antibody (rabbit monoclonal)	CST #3195S	α-ECad	1:500
Antibody	Anti-N-Cadherin Antibody (rabbit monoclonal)	CST #13116S	α-NCad	1:200
Antibody	Anti-HSC70 Antibody (mouse monoclonal)	Santa Cruz Biotechnology	α-HSC70	1:1,000
Antibody	IRDye anti-rabbit 800 (donkey polyclonal)	Licor #925-32212		1:10,000
Antibody	IRDye anti-mouse 680 (goat polyclonal)	Licor #925-68070		1:10,000
Antibody	anti-Rabbit IgG Alexa Fluor 405 (donkey polyclonal)	Abcam #175649		1:400
Software, algorithm	CellProfiler	https://cellprofiler.org/		
Software, algorithm	findPeaks matlab script	T. C. O’Haver, 2014; Mathworks.com		modified to detect rate of change between gaussian-fitted minima and maxima from single cell traces
Software, algorithm	Proteome Discoverer	Thermo Fisher, v2.3		
Software, algorithm	Mascot	Matrix Science, v2.6.2		
Software, algorithm	preprocessImagesCaller.py	Aikin T., Peterson A., Pokrass M., Clark H., Regot S., PreprocessImagesCaller. GitHub. https://github.com/tjaikin/Regot-Lab/blob/Aikin_2020/preprocessImagesCaller.py. dc08aeb.		
Software, algorithm	preprocessImages.py	Aikin T., Peterson A., Pokrass M., Clark H., Regot S., PreprocessImages. GitHub. https://github.com/tjaikin/Regot-Lab/blob/Aikin_2020/preprocessImages.py. dc08aeb.		
Software, algorithm	flatfielding.py	Aikin T., Peterson A., Pokrass M., Clark H., Regot S., Flatfielding. GitHub. https://github.com/tjaikin/Regot-Lab/blob/Aikin_2020/flatfielding.py.dc08aeb.		
Software, algorithm	registerAndCrop.py	Aikin T., Peterson A., Pokrass M., Clark H., Regot S., registerAndCrop. GitHub. https://github.com/tjaikin/Regot-Lab/blob/Aikin_2020/registerAndCrop.py.dc08aeb.		
Software, algorithm	trackOrganizeCpDataCaller.py	Aikin T., Peterson A., Pokrass M., Clark H., Regot S., trackOrganizeCpDataCaller. GitHub. https://github.com/tjaikin/Regot-Lab/blob/Aikin_2020/trackOrganizeCpDataCaller.py.dc08aeb.		
Software, algorithm	trackOrganizeCpData.py	Aikin T., Peterson A., Pokrass M., Clark H., Regot S., PreprocessImages. GitHub. https://github.com/tjaikin/Regot-Lab/blob/Aikin_2020/trackOrganizeCpData.py.dc08aeb.		
