# Supplementary material for: MAPK activity dynamics regulate non-cell autonomous effects of oncogene expression
Source: eLife. 2020 Sep 17;9:e60541. doi: 10.7554/eLife.60541 (PMC7498266; doi:10.7554/eLife.60541)
Supplement: Supplementary file 1. — Supernatants from ADAM17KO or WT cells expressing (+Dox) or not expressing (-Dox) BRAFV600E were collected and analyzed by Tandem-Mass-Tag (TMT) mass spectrometry as described in methods. Table shows fold change and p-values for the 24 identified factors presented in Figure 3D in red. [file elife-60541-supp1.pdf]

Supplementary Table 1

Secreted Factor	+Dox. / -Dox.		WT / ADAM17-KO	
	Fold Change	P-value	Fold Change	P-value
AREG	8.35	5.88E-03	10.75	4.39E-03
SDC4	5.32	9.18E-03	4.52	3.90E-03
NECTIN1	3.67	1.49E-03	4.67	3.92E-03
APP	3.25	4.29E-03	1.98	1.03E-02
PTPRS	2.83	1.42E-02	3.23	2.18E-03
PIK3IP1	2.83	2.89E-02	1.80	4.12E-02
JAG1	2.75	1.24E-02	2.85	2.28E-03
DDR1	2.69	2.37E-02	2.36	7.74E-03
DAG1	2.67	1.73E-02	2.16	1.27E-02
SFRP1	2.60	3.75E-02	2.70	3.69E-02
NEO1	2.53	1.02E-02	2.56	8.18E-03
MDK	2.51	3.16E-02	1.98	1.54E-02
HLA-C	2.48	1.62E-02	1.99	2.17E-02
MET	2.22	3.56E-02	2.21	1.60E-02
PTPRF	2.20	1.10E-02	2.36	1.87E-03
SDC1	2.16	3.73E-02	1.61	4.11E-02
MSLN	2.15	1.28E-02	1.59	4.60E-02
SPINT1	2.10	1.93E-02	1.83	1.90E-02
HLA-B	2.07	1.23E-02	2.87	9.49E-03
PTK7	2.05	3.81E-02	3.37	3.25E-03
APLP2	1.91	7.05E-03	1.93	1.47E-03
PROCR	1.87	4.70E-02	2.96	4.26E-02
HLA-A	1.71	2.05E-02	2.22	1.05E-02
CCT3	1.54	1.51E-03	1.65	1.07E-02
