# Supplementary material for: MAPK activity dynamics regulate non-cell autonomous effects of oncogene expression
Source: eLife. 2020 Sep 17;9:e60541. doi: 10.7554/eLife.60541 (PMC7498266; doi:10.7554/eLife.60541)
Supplement: Transparent reporting form [file elife-60541-transrepform.pdf]

eLIFE
Westbrook Centre, Milton Road
Cambridge CB4 1YG
UK
P 01223 855340
W elifesciences.org
T @elife

eLife's transparent reporting form

We encourage authors to provide detailed information *within their submission* to facilitate the interpretation and replication of experiments. Authors can upload supporting documentation to indicate the use of appropriate reporting guidelines for health-related research (see EQUATOR Network), life science research (see the BioSharing Information Resource), or the ARRIVE guidelines for reporting work involving animal research. Where applicable, authors should refer to any relevant reporting standards documents in this form.

If you have any questions, please consult our Journal Policies and/or contact us: editorial@elifesciences.org.

Sample-size estimation

- You should state whether an appropriate sample size was computed when the study was being designed
- You should state the statistical method of sample size computation and any required assumptions
- If no explicit power analysis was used, you should describe how you decided what sample (replicate) size (number) to use

Please outline where this information can be found within the submission (e.g., sections or figure legends), or explain why this information doesn't apply to your submission:

For imaging experiments, see methods "Live Imaging". For mass spectrometry, "triplicate samples" were collected and analyzed as outlined in the methods "Proteomics". For cell proliferation assays, see methods "Cell Proliferation Assay". In directed migration experiments, sample sizes were vey large, and we subjected data to subsampling methods before testing, see methods "Image Analysis and Quantification". For extrusion experiments, see methods "Image Analysis and Quantification".

Replicates

- You should report how often each experiment was performed
- You should include a definition of biological versus technical replication
- The data obtained should be provided and sufficient information should be provided to indicate the number of independent biological and/or technical replicates
- If you encountered any outliers, you should describe how these were handled
- Criteria for exclusion/inclusion of data should be clearly stated
- High-throughput sequence data should be uploaded before submission, with a private link for reviewers provided (these are available from both GEO and ArrayExpress)

Please outline where this information can be found within the submission (e.g., sections or figure legends), or explain why this information doesn't apply to your submission:

For imaging experiments, Inclusion criteria and cleaning is described in methods "Live Imaging". For mass spectrometry, "triplicate samples" were used. For cell proliferation assays, see methods "Cell Proliferation Assay". For extrusion experiment inclusion criteria, see methods "Image Analysis and Quantification".

eLife Sciences Publications, Ltd is a limited liability non-profit non-stock corporation incorporated in the State of Delaware, USA, with company number 5030732, and is registered in the UK with company number FC030576 and branch number BR015634 at the address Westbrook Centre, Milton Road Cambridge CB4 1YG, UK | March 2019

Statistical reporting

- Statistical analysis methods should be described and justified
- Raw data should be presented in figures whenever informative to do so (typically when N per group is less than 10)
- For each experiment, you should identify the statistical tests used, exact values of N, definitions of center, methods of multiple test correction, and dispersion and precision measures (e.g., mean, median, SD, SEM, confidence intervals; and, for the major substantive results, a measure of effect size (e.g., Pearson's r, Cohen's d)
- Report exact p-values wherever possible alongside the summary statistics and 95% confidence intervals. These should be reported for all key questions and not only when the p-value is less than 0.05.

Please outline where this information can be found within the submission (e.g., sections or figure legends), or explain why this information doesn't apply to your submission:

For mass spectrometry, see the methods "Proteomics" and the legend for Figure 3D and F3-S1. For cell proliferation assays, see methods "Cell Proliferation Assay" and figure legends for sample sizes. In directed migration experiments, sample sizes were vey large, and we subjected data to subsampling methods before testing, see methods "Image Analysis and Quantification" and figure legends for sample sizes. For extrusion experiments, see methods "Image Analysis and Quantification" and figure legends for sample sizes. P-values are given throughout the figures as follows: "ns" not significant, * p<0.05, ** p<0.01, *** p<0.001.

(For large datasets, or papers with a very large number of statistical tests, you may upload a single table file with tests, Ns, etc., with reference to sections in the manuscript.)

Group allocation

- Indicate how samples were allocated into experimental groups (in the case of clinical studies, please specify allocation to treatment method); if randomization was used, please also state if restricted randomization was applied
- Indicate if masking was used during group allocation, data collection and/or data analysis

Please outline where this information can be found within the submission (e.g., sections or figure legends), or explain why this information doesn't apply to your submission:

Image analysis is handled automatically via an identical pipeline as described in methods "Image Analysis and Quantification"

Additional data files ("source data")

- We encourage you to upload relevant additional data files, such as numerical data that are represented as a graph in a figure, or as a summary table
- Where provided, these should be in the most useful format, and they can be uploaded as "Source data" files linked to a main figure or table
- Include model definition files including the full list of parameters used
- Include code used for data analysis (e.g., R, MatLab)
- Avoid stating that data files are "available upon request"

Please indicate the figures or tables for which source data files have been provided:

Source Data for identified secreted factors in mass spectrometry (Fig. 3D) appears as a figure supplement table. For image analysis, we use CellProfiler and several custom scripts deposited in GitHub and referenced in the Key Resources Table. For Peak counting, we use findPeaks, also listed in the Key Resources.
