# Supplementary material for: MAPK activity dynamics regulate non-cell autonomous effects of oncogene expression
Source: eLife. 2020 Sep 17;9:e60541. doi: 10.7554/eLife.60541 (PMC7498266; doi:10.7554/eLife.60541)
Supplement: Peer review history

# Decision letter

**DOI:** 10.7554/eLife.60541.sa1

**Affiliations:** King's College LondonUnited Kingdom; King's College LondonUnited Kingdom

In the interests of transparency, eLife publishes the most substantive revision requests and the accompanying author responses.

**Acceptance summary:**

This work highlights how pulsed vs. sustained ERK signalling differentially effect cell migration, proliferation, and elimination by imaging single cell signaling within epithelial monolayers. While pulsatile ERK signalling causes cell proliferation, sustained ERK signals lead to cycle arrest and elimination by Epithelial Defense Against Cancer, where oncogenic cells are extruded. This new ability to visualize single cell signals shows how ERK causes differential effects on both oncogenic and nearby cells to coordinate their removal and replacement.

**Decision letter after peer review:**

[Editors’ note: the authors submitted for reconsideration following the decision after peer review. What follows is the decision letter after the first round of review.]

Thank you for submitting your work entitled "Collective MAPK signaling dynamics coordinates epithelial homeostasis" for consideration by *eLife*. Your article has been reviewed by three peer reviewers, one of whom is a member of our Board of Reviewing Editors, and the evaluation has been overseen by a Senior Editor. The reviewers have opted to remain anonymous.

Our decision has been reached after consultation between the reviewers. Based on these discussions and the individual reviews below, we regret to inform you that your work will not be considered for publication in *eLife* in its current state. While you are welcome to send it elsewhere, we would like to give you an option to resubmit it as a new submission and I will try to get the same reviewers to address your changes.

While you will see that all the reviewers felt that the level of data was high, we all felt the main problem with the manuscript in its current form is that it overstates what the data says. I, myself, felt that the manuscript lacked a simple overarching finding. Instead, it was many findings, culminating with a title that is so general that we don't actually understand a clear take-home message. Indeed, we even felt you could publish in its current form (possibly) if rewritten entirely to only make claims that are actually supported by the data. While we do not want to prescribe how you might rewrite it, I think it would be helpful if you to state the questions and then answer each with solid data (without overreaching). Additionally, we felt that some data needs to actually be shown, especially if it is mentioned in the Abstract, on “extrusion”. There are different ways to delaminate cells and the signaling that does so is different, therefore, we need to know if what you are describing as extrusion is the (Piezo1)-S1P-Rho-actomyosin one or the Epithelial Defense against Cancer one or simply elongation of cells in a pseudostratification, each of which have different cell biological hallmarks. We hope that the following complete reviews will help you with your decision on how you might revise this or submit elsewhere.

Reviewer #1:

Aikin et al. combine biosensors, inducible oncogene expression systems and an impressive live-imaging and analytical capacity to study the early effects of oncogenic signalling on ERK activity (pulses/sustained) and location (nuclear/cytoplasmic), and how these differently affect migration, proliferation, and extrusion in epithelial monolayers. Oncogene-driven sustained ERK activation promotes mutant cell cycle arrest and ADAM17-mediated growth factor shedding. Through paracrine signalling, this increases ERK waves in oncogene-free neighbouring cells to promote their proliferation. Moreover, these waves orient wild type cell migration towards the oncogene-expressing cell, resulting in its extrusion. This is an interesting study that highlights the relevance of cell signalling and the resulting collective dynamics in tissue homeostasis.

1) While this is an interesting foray into how cells differentially expressing different oncogenes interact within a monolayer, the story as a whole, lacks a coherent, unifying message. This could be addressed by addition of a schematic summarizing the findings, with colour coding representing different types of ERK waves. It could also use some rationale for why different signals might behave in such polarizing ways. At the moment the work is interesting but feels like a bunch of separate stories that do not fit a thematic story.

2) While the authors refer to extrusion throughout the text, there is no data within the paper supporting that the cells extrude through the classical mechanisms published, actomyosin ring assembly. Indeed, it seems that extrusion here does not require S1P, which it does in canonical extrusion studies. Might this be like the HRas and Src studies that are eliminated by a similar mechanism, EDAC? They are using MCF10A, which do not form proper junctions unless grown in cysts, so perhaps this is not extrusion that is driven by epithelia that make tight junctions to each other. It is important to learn if the signalling they have identified is really controlling bonified extrusion or something like it.

Reviewer #2:

In this article, Aikin and colleagues describe how epithelial cells propagate ERK activity waves in pathological conditions: i.e. in presence of a tumor subpopulation or in acute UV-mediated injury. Methodologically, they combined fluorescent reporters of Erk activity and Erk localization, an oncogene inducible system, time-lapse microscopy and computer-vision image analysis. Using these tools, the authors found that oncogene induction in an epithelial subpopulation induces a change in Erk signalling dynamics and Erk localization in the oncogene-induced cells but also in the neighbors. They identified an AREG-EGFR paracrine cell-cell communication system responsible for the activation of Erk dynamics into the neighbors. Such aberrant communication between oncogene-induced and neighboring cells in responsible for the extrusion of oncogene-induced cells and increased proliferation in the neighbors. In addition, they identified that the same Erk activity propagation happens from UV-stressed epithelium into the neighboring healthy cells.

The data is of high quality, and the findings are clearly important for the cancer signaling community. However, a lot of the data is strongly overinterpreted, and I think that the authors have to make a large effort to rethink what they can conclude and not conclude from their data. The authors did not focus particularly on one of the many mechanisms they document to provide clear mechanistic details. In the Discussion, the results are interpreted in light of a mix of cell competition, epithelial homeostasis, signaling dynamics conceptual frameworks but no clear mechanistical explanations emerges for some of the (very) strong claims the authors make. Also, very often, the authors make some very strong statements without going into the details of what they want to say (just providing some references). This caused quite some confusion for me while reading the paper. In my opinion the paper should be rewritten completely to be appropriate for *eLife* (I do not have problems with the data). Also, the authors have to make additional experiments if they want to stick to the strong claims they make (or simply tone down the interpretation of the results).

1) The authors propose that the different cell cycle entry versus motility are solely induced by the different patterns of ERK activity (pulsatile versus sustained)! This is not compatible with the view that different oncogenic mutations can trigger a large number of pathway: (1) EGFR controls MAPK, PI3K and PLC; (2) B-RAF crosstalks with ROCK (see the work of the Baccarini lab).

2) The authors make a strong case that the temporal patterns of ERK activity in the cancer cells are decoded by a paracrine growth factor signaling mechanism leading to ERK waves in the healthy cells. Despite this strong claim, the authors do not provide a mechanistic explanation about how this happens. It is not clear to me how healthy cells can decode a sustained ERK signaling state in the cancer cells, and if ERK dynamics is the feature that switches ON paracrine signaling/ERK waves in the surrounding healthy cells?

3) Rather, the BRAF^V600E^ cells and MEK2^DD^ cells have clearly undergone an EMT (clearly visible in the Video 1) and are thus might be much less adhesive. I think that this can provide a simple explanation how they can be detected/extruded by healthy cells in the monolayer. If the authors want a causal link between mutation state and paracrine signaling, they should rather work towards that EMT concept (e.g. checking adhesive state of the cells – E-cadherin versus N-Cadherin expression, restoring adhesion, understanding how sustained ERK activity leads to EMT/increased motility – I am sure there must be abundant literature about ERK and motility).

4) Overexpression of oncogenes has been shown to lead to different phenotypes than knockin of these oncogenes that better mimic the (especially evident for the Ras pathway). This should be strongly emphasized in the Discussion.

5) The title is misleading and overreaching. There isn't any single experiment to investigate the role of ERK signalling dynamics in epithelial homeostasis conditions (e.g. in absence of cancer mutations). Rather, the authors investigated the propagation of MAPK activation from two pathological conditions (i.e. oncogene-induced subpopulation and acute UV-induced injury).

6) EGFR-ligands/EGFR communicates the Erk signalling pulses to the neighboring cells. However, it doesn't prove that AREG is the only EGFR ligand involved in such communication. I suggest the author to specifically block this ligand with an AREG neutralizing antibody or to knock-down AREG to prove the specificity of the pathway.

7) The expression levels of BRAF seems to have a strong effect on signalling. For instance, in Figure 1C BRAF^WT^ overexpression induces a significant increase of the number of Erk pulses. It is not clear to me how the authors can distinguish the effects of the V600E mutation from the simple BRAFoverexpression. How could the author be sure that the expression of BRAF^WT^ and BRAF^V600E^ are comparable in Figure 2C and other experiments? The use of a V600E-specific inhibitor in the experiments shown if Figures 2 and 3 would help to distinguish between the effects of overexpression and mutation.

8) The extrusion assay based on nuclear position shown throughout the paper is practical and informative, however doesn't distinguish between elongated or actually extruded cells. For instance, it could be that cells acquire an elongated morphology, while maintaining contact with the substrate. I recommend the authors to use a cytoplasm or membrane staining to prove that cells are really extruded and not just elongated or pseudostratified. E-Cadherin should be visualized to test if the cancer mutations induce EMT, and if this leads to lower adhesion of the cancer cells to the healthy monolayer.

Reviewer #3:

The manuscript by Aikin et al. demonstrates that two different patterns of ERK activity lead to distinct cellular outcomes. They have used the ERK signaling biosensor to detect ERK activity patterns in single cells and found that sustained ERK activity induced by the oncogene expression or UV exposure results in EGFR ligand secretions shed by ADAM17. The ADAM17-EGFR signaling triggers ERK waves in the neighboring cells, thereby promoting their polarized movement and extrusion of oncogenic or stressed cells in a non-cell autonomous manner. Overall, most of the experiments are properly performed, and the data are of high quality. I feel that this study is thus potentially suitable for *eLife*. However, some of the conclusions are not sufficiently supported by the presented data, thus the authors should address the following concerns before publication.

ERK waves have been demonstrated and analyzed in previous studies. The novel finding shown in this study is that ERK waves around transformed or apoptotic cells induce the polarized movement of the surrounding cells and promote cell extrusion. To consolidate this finding, the following concern should be addressed. In its current form, some of the authors' conclusions fall into overstatement.

1) The authors describe that “activation of inducible cells alone is not sufficient for extrusion, and neighboring cell EGFR-ERK activation is required”. However, they have only tested the effect of inhibitors. The authors should establish EGFR-knockdown or knockout cells and examine the effect of EGFR-depletion in the surrounding cells on ERK activation, cell movement and cell extrusion.

2) How ERK waves induce the polarized movement of the surrounding cells? The link between the two processes is not studied or described in this study at all. If the authors' conclusion is right, secreted EGFR ligands bind to EGFR on the apical surface of the surrounding epithelial cells. It is hard to imagine (believe) whether and how epithelial cells sense the direction of extruding cells from this input and generate the planar polarity. The authors should make some efforts to link the two processes. Any planar polarized molecules or structures after ERK waves (e.g. centrosomes or Golgi)?

[Editors’ note: further revisions were suggested prior to acceptance, as described below.]

Thank you for submitting your article "MAPK Activity Dynamics Regulate Non-Cell Autonomous Effects of Oncogene Expression" for consideration by *eLife*. Your article has been reviewed by three peer reviewers, including Jody Rosenblatt as the Reviewing Editor and Reviewer #1, and the evaluation has been overseen by Jonathan Cooper as the Senior Editor.

The reviewers have discussed the reviews with one another and the Reviewing Editor has drafted this decision to help you prepare a revised submission.

We would like to draw your attention to changes in our revision policy that we have made in response to COVID-19 (https://elifesciences.org/articles/57162). Specifically, we are asking editors to accept without delay manuscripts, like yours, that they judge can stand as *eLife* papers without additional data, even if they feel that they would make the manuscript stronger. Thus the revisions requested below only address clarity and presentation.

Summary:

The manuscript by Aikin et al. highlight how pulsed vs sustained ERK signalling differentially effect cell migration, proliferation, and elimination in mammalian epithelial monolayers. Using live imaging of biosensors and inducible oncogenes, they reveal that mutations resulting in pulsatile ERK signalling lead to mutant cell proliferation, while mutations resulting in sustained ERK signals lead to their cycle arrest and elimination by Epithelial Defense Against Cancer. Sustained ERK causes ADAM17-mediated AREG shedding, inactivating EGFR, but increasing ERK waves in oncogene-free neighbouring cells, promoting their cycle progression and oriented cell migration towards the oncogene-expressing cell.

Revisions:

We have found the revised manuscript much improved and, while all reviewers are in favor of publication now, we would like you to edit it for clarity and to highlight a few points and secure some statistical analysis. I will leave below the specific comments below for your reference. In general, two of us felt that the discussion on “extrusion” was not all that clear and that your data are more supportive of EDAC so that this should be clarified throughout, not supported by disrupting actin dynamics or showing a clear ring. While we do not request more experiments, we think it is important to not confuse the audience, as EDAC and Extrusion are controlled by different signaling and mechanisms and we need to be clear on what signaling drives each. Additionally, I felt the manuscript would benefit a bit by more clear and concise language, especially in sections that are most frequently read, such as the Abstract, last paragraph of Introduction, and the first paragraph of the Discussion. Therefore, I offered a few suggestions to tighten up the text. I liked the schematic and felt that it serves as a great talking point for the Abstract and Discussion.

Reviewer #1

Several articles have recently highlighted differential outcomes of pulsatile vs sustained ERK signalling and their relevance to homeostasis versus transformation, making this addition to the field timely. We feel that the authors have greatly improved their data since the last submission and are in favour of its acceptance, once they address the following concerns.

1) The Abstract describes the work in a very diffuse way, not concisely stating what the results are. One should be able to understand what the take-home message of the paper is saying from the Abstract. I think that the Nature website gives a good guide on how to write a useful Abstract. It is important to note that most people will only ever read this. Relay exactly what your paper's results are, i.e. exactly what you find in Figure 8.

2) The same is true in your Discussion. The first paragraph should be a nice recap of your findings that points to Figure 8. Instead that first paragraph is vague and difficult to follow. The last paragraph should be more of a zoom out to talk about the impact of this work in general, instead of just repeating what you have said several times.

3) The authors have addressed the previous concerns by adding a substantial amount of data, but some quantifications (e.g. radial histograms in Figures 5, 6, 7) still lack statistical analysis.

Reviewer #2:

In the revised manuscript, the authors have responded to most of my comments properly, and the paper has been substantially improved. This study is, in principle, acceptable for *eLife*.

Reviewer #3:

In this revision of the paper by Aikin et al. about non-autonomous ERK signalling dynamics and implications in cell-extrusion and non-autonomous proliferation, the authors revised the manuscript according to my and other reviewer's suggestions. The manuscript is now more linear and better supported by additional experiments. In particular, the authors provided a better characterization of the extrusion process induced by oncogene expression, about EMT, and the role of S1P and AREG during extrusion. Moreover, they provided additional controls about the expression levels of WT and mutated BRAF, as well as a dose-response curve for Gefitinib. In my opinion the manuscript is much improved, and could be published without the need of any additional experiments.

# Reply: Author response

**DOI:** 10.7554/eLife.60541.sa2

[Editors’ note: the authors resubmitted a revised version of the paper for consideration. What follows is the authors’ response to the first round of review.]

Reviewer #1:

Aikin et al. combine biosensors, inducible oncogene expression systems and an impressive live-imaging and analytical capacity to study the early effects of oncogenic signalling on ERK activity (pulses/sustained) and location (nuclear/cytoplasmic), and how these differently affect migration, proliferation, and extrusion in epithelial monolayers. Oncogene-driven sustained ERK activation promotes mutant cell cycle arrest and ADAM17-mediated growth factor shedding. Through paracrine signalling, this increases ERK waves in oncogene-free neighbouring cells to promote their proliferation. Moreover, these waves orient wild type cell migration towards the oncogene-expressing cell, resulting in its extrusion. This is an interesting study that highlights the relevance of cell signalling and the resulting collective dynamics in tissue homeostasis.

1) While this is an interesting foray into how cells differentially expressing different oncogenes interact within a monolayer, the story as a whole, lacks a coherent, unifying message. This could be addressed by addition of a schematic summarizing the findings, with colour coding representing different types of ERK waves. It could also use some rationale for why different signals might behave in such polarizing ways. At the moment the work is interesting but feels like a bunch of separate stories that do not fit a thematic story.

We agree with the reviewer and have now implemented the following changes:

1) We have sharpened our message by specifically addressing the role of ERK pathway oncogene expression on signaling dynamics and resulting cell behaviors by rewriting the text and rearranging the main figures.

2) We have removed separate findings about UV-induced apoptosis that were distracting of the central message.

3) We have added a summary schematic (Figure 8) to illustrate how single cell ERK dynamics lead to specific autonomous and non-cell autonomous behaviors.

4) We speculate on the mechanistic basis of dynamics-specific paracrine signaling. Previous studies showed that ADAM17 is a weaker ERK substrate than ELK, thus sustained activity may be required to accumulate active ADAM17 (Results paragraph five).

2) While the authors refer to extrusion throughout the text, there is no data within the paper supporting that the cells extrude through the classical mechanisms published, actomyosin ring assembly. Indeed, it seems that extrusion here does not require S1P, which it does in canonical extrusion studies. Might this be like the HRas and Src studies that are eliminated by a similar mechanism, EDAC? They are using MCF10A, which do not form proper junctions unless grown in cysts, so perhaps this is not extrusion that is driven by epithelia that make tight junctions to each other. It is important to learn if the signalling they have identified is really controlling bonified extrusion or something like it.

We performed several experiments to relate the extrusion observed here to the processes of delamination, cell elongation, and apoptotic or EDAC extrusion (Figure 5 and Figure 5—figure supplement 2):

1) We show that oncogenic cells are apically extruded (not elongated) and maintain E-Cad expression (not delaminated).

2) We used live imaging of actin dynamics using Utrophin-261-EGFP and showed polarized actin enrichment in neighboring cells.

3) Since our system seems to be only partially affected by S1P inhibitors we agree with the reviewer that the process observed resembles EDAC more than extrusion. Thus, we have changed the text accordingly.

In studying the effects of S1P in these processes we made an interesting observation: blocking S1P production had no effect on the signaling, migration, or extrusion of MEK2^DD^ cells (Figure 5—figure supplement 2E-G). However it did have an effect on extrusion in MKK3^DD^ cells (Figure 6—figure supplement 4). We attribute the different requirement for S1P production to a difference in whether extruded cells have either active ERK (with increased migration) or active p38 (Figure 6—figure supplement 1C). These observations are summarized in the Results and Discussion.

Reviewer #2:

[…]

1) The authors propose that the different cell cycle entry versus motility are solely induced by the different patterns of ERK activity (pulsatile versus sustained)! This is not compatible with the view that different oncogenic mutations can trigger a large number of pathway: (1) EGFR controls MAPK, PI3K and PLC; (2) BRAF crosstalks with ROCK (see the work of the Baccarini lab).

We acknowledge that oncogenes at different levels will activate different downstream pathways likely having different phenotypic consequences. However, our results show that independently of what signaling node is perturbed in the cascade the temporal patterns of ERK activity (sustained or pulsatile) were always correlated to the same phenotypes (cell cycle arrest with paracrine signaling or cellautonomous proliferation respectively). These data provide correlative evidence linking ERK dynamics to cell behavior. The text has been modified to indicate the correlative nature of these observations.

In particular, if BRAF^V600E^ effects were a result of BRAF-ROCK crosstalk through RAF1 (as demonstrated by the Baccarini lab), ROCK activation would be absent in the case of sustained ERK activity after MEK2^DD^ induction. Yet BRAF^V600E^ and MEK2^DD^ both lead to increased migration and decreased proliferation. Similarly, if EGFR acted through the alternate pathways mentioned (PI3K, PLC) to elicit increased proliferation, that crosstalk would be absent during BRAF^WT^ induction. Thus cell behavior correlated with ERK dynamics even though the different perturbations are likely to activate different downstream pathways.

We highlight the comparison of BRAF^WT^ and BRAF^V600E^ because they are nearly identical proteins that show qualitatively different dynamics yet opposed cellular behaviors. The potential mechanisms behind these different downstream ERK dynamics are now discussed.

2) The authors make a strong case that the temporal patterns of ERK activity in the cancer cells are decoded by a paracrine growth factor signaling mechanism leading to ERK waves in the healthy cells. Despite this strong claim, the authors do not provide a mechanistic explanation about how this happens. It is not clear to me how healthy cells can decode a sustained ERK signaling state in the cancer cells, and if ERK dynamics is the feature that switches ON paracrine signaling/ERK waves in the surrounding healthy cells?

We apologize for the confusion. We have tried to better explain the proposed mechanism for paracrine signaling in the text and figures, and have provided a graphical summary (see Figure 7).

Briefly, we show that inducing sustained ERK activity in a subset of cells results in ERK activity waves through the surrounding monolayer. This process is ADAM17, AREG and EGFR dependent. Thus, we propose that sustained ERK activity is decoded by activation of ADAM17 within the oncogenic cells. ADAM17 then cleaves AREG from the membrane to diffuse and engage EGF receptors on neighboring cells. ADAM17 paracrine signaling is dynamics-dependent since it only occurs upon sustained ERK activity (See Figure 2, Figure 3G and Figure 7).

We also propose a mechanism for how ADAM17 activation may depend on the temporal patterns of ERK activity. Previous studies showed that phosphorylation of ADAM17 by ERK is weaker than ELK, thus if dephosphorylation occurs at the same rate, sustained activity may be required to accumulate active ADAM17 at meaningful levels.

3) Rather, the BRAF^V600E^ cells and MEK2^DD^ cells have clearly undergone an EMT (clearly visible in the Video 1) and are thus might be much less adhesive. I think that this can provide a simple explanation how they can be detected/extruded by healthy cells in the monolayer. If the authors want a causal link between mutation state and paracrine signaling, they should rather work towards that EMT concept (e.g. checking adhesive state of the cells – E-cadherin versus N-Cadherin expression, restoring adhesion, understanding how sustained ERK activity leads to EMT/increased motility – I am sure there must be abundant literature about ERK and motility).

We thank the reviewer for these insightful comments. We agree that the rapid migration and loss of contact observed following induction of BRAF^V600E^ and MEK2^DD^ in Video 1 are reminiscent of EMT.

1) To directly address the role of EMT in these contexts, we performed immunofluorescent staining for E-Cadherin (E-Cad) and N-Cadherin (N-Cad) and compared induced cells to cells treated with TGF-b to induce EMT (Figure 1—figure supplement 3). We did not find loss of E-Cad expression in the cases of BRAF^V600E^ and MEK2^DD^ induction, and N-Cad expression did not compare to the levels observed in EMT’d cells. We conclude that “results indicate that at the time points studied here, altered cell behaviors are either distinct from or precede those resulting from EMT”.

2) E-Cad immunofluorescence helped us address the reviewer’s question about loss of adhesion. Our experiments show that single oncogenic cells maintain E-Cad expression in coculture, and E-Cad is enriched at the boundaries with neighboring cells. We have presented this finding in the text and figures (Figure 5—figure supplement 2A).

4) Overexpression of oncogenes has been shown to lead to different phenotypes than knockin of these oncogenes that better mimic the (especially evident for the Ras pathway). This should be strongly emphasized in the Discussion.

The reviewer is correct about this distinction, and our results are now discussed alongside mention of this caveat, reading:

“While our approach is admittedly different than acquisition of point mutations in vivo, ERK dynamics resulting from oncogene overexpression robustly correlated with cellular phenotypes.”

In addition, we have included a western blot showing expression of BRAF inducible constructs compared to WT cells (Figure 1—figure supplement 2).

5) The title is misleading and overreaching. There isn't any single experiment to investigate the role of ERK signalling dynamics in epithelial homeostasis conditions (e.g. in absence of cancer mutations). Rather, the authors investigated the propagation of MAPK activation from two pathological conditions (i.e. oncogene-induced subpopulation and acute UV-induced injury).

We modified the title in order to reflect the restructured focusing of the text on ERK signaling dynamics specifically in the oncogenic context.

To support this new focus, we eliminated data and text related to the response to UV-light. We kept a single video documenting similar ERK waves following spontaneous cell death (Figure 2—video 2 ), because we hypothesize that the waves occur via similar mechanisms to facilitate a similar protective effect, as has been suggested by two recent preprints since the submission of our work (see Gagliardi et al., 2020; Valon et al., 2020; Results and Discussion).

6) EGFR-ligands/EGFR communicates the Erk signalling pulses to the neighboring cells. However, it doesn't prove that AREG is the only EGFR ligand involved in such communication. I suggest the author to specifically block this ligand with an AREG neutralizing antibody or to knock-down AREG to prove the specificity of the pathway.

We thank the reviewer for suggesting the use of function blocking antibodies (FB Ab’s) against AREG. We treated 10% MEK2^DD^ cocultures with AREG FB Ab’s and they reduced paracrine signaling to the neighboring cells. This result is now presented in Figure 3F.

7) The expression levels of BRAF seems to have a strong effect on signalling. For instance, in Figure 1C BRAF^WT^ overexpression induces a significant increase of the number of Erk pulses. It is not clear to me how the authors can distinguish the effects of the V600E mutation from the simple BRAF overexpression. How could the author be sure that the expression of BRAF^WT^ and BRAF^V600E^ are comparable in Figure 2C and other experiments? The use of a V600E-specific inhibitor in the experiments shown if Figures 2 and 3 would help to distinguish between the effects of overexpression and mutation.

We thank the reviewer for this insightful comment. We have analyzed the expression level of BRAF^WT^ to BRAF^V600E^ by immunoblotting and found them to be similarly overexpressed when compared to the uninduced controls (Figure 1—figure supplement 2). We believe that since these we use these expression systems only to alter downstream ERK signaling dynamics in a temporally controlled manner, differentiation between the effects of overexpression alone or activity+overexpression is beyond the scope of the current study.

8) The extrusion assay based on nuclear position shown throughout the paper is practical and informative, however doesn't distinguish between elongated or actually extruded cells. For instance, it could be that cells acquire an elongated morphology, while maintaining contact with the substrate. I recommend the authors to use a cytoplasm or membrane staining to prove that cells are really extruded and not just elongated or pseudostratified. E-Cadherin should be visualized to test if the cancer mutations induce EMT, and if this leads to lower adhesion of the cancer cells to the healthy monolayer.

We thank the reviewer for this insightful comment. As discussed in the response to major comment 3, ECad and N-Cad staining did not indicate that oncogenic cells underwent EMT by the time of extrusion.

The reviewer accurately assesses the disadvantages of using nuclear position to measure extrusion. To rigorously test whether cells were elongated, delaminated, or fully extruded, we looked at E-Cad immunostaining of oncogenic cells in coculture. This assay revealed that (i) BRAF^V600E^ cells maintain ECad expression, (ii) that E-Cad is enriched at sites of contact with neighboring cells, and (iii) that the majority (91%) of oncogenic cells are extruded by 24 hours, sitting on top of other cells without any basal attachment. These results are presented in the text and figures (Figure 5—figure supplement 2A-B).

Finally, we used Utrophin-261-EGFP to observe actin dynamics during extrusion (see reviewer 1 major comment 2). These images show complete eclipsing of any basal attachment of an oncogenic cell by its neighbors (Figure 5—figure supplement 2C and Video 4).

Reviewer #3:

[…]

1) The authors describe that “activation of inducible cells alone is not sufficient for extrusion, and neighboring cell EGFR-ERK activation is required”. However, they have only tested the effect of inhibitors. The authors should establish EGFR-knockdown or knockout cells and examine the effect of EGFR-depletion in the surrounding cells on ERK activation, cell movement and cell extrusion.

We thank the reviewer for this thoughtful comment. Throughout the manuscript we cite previous work characterizing the mechanisms of spontaneous ERK waves in the skin, and during collective migration following scratch depending on ADAM17 and EGFR signaling. To our knowledge, ERK activity waves have not been observed in the context of oncogenesis, and we discovered that the waves are MAPK dynamics-dependent (depend on sustained ERK activity or p38 activity in single cells).

Knockout of EGFRs is technically challenging because MCF10A cells rely on EGFR signals for growth and survival, and because a family of potentially redundant EGFR receptors would have to be knocked down simultaneously. We now acknowledge in the text that experiments with EGFR inhibitor cannot discriminate between inducible or neighboring cells.

Regarding the requirement of ERK activation specifically in neighboring cells, we use p38-active cells to provide an ERK-independent stimulation of ADAM17-mediated shedding. In this context we can inhibit ERK without interrupting the generation of paracrine signals. By blocking the activation of neighboring cells (in MKK3^DD^ cocultures pretreated with MEK or EGFR inhibitor), directed migration and extrusion are both significantly reduced. These results are presented in Figure 6 and discussed in the text.

2) How ERK waves induce the polarized movement of the surrounding cells? The link between the two processes is not studied or described in this study at all. If the authors' conclusion is right, secreted EGFR ligands bind to EGFR on the apical surface of the surrounding epithelial cells. It is hard to imagine (believe) whether and how epithelial cells sense the direction of extruding cells from this input and generate the planar polarity. The authors should make some efforts to link the two processes. Any planar polarized molecules or structures after ERK waves (e.g. centrosomes or Golgi)?

We thank the reviewer for this question, which led to an interesting set of experiments.

We have now grouped evidence supporting the directional information properties (or “coordination” role) of growth factor paracrine signaling into a new figure (Figure 7), and emphasized these results in the Discussion. To disrupt spatially-organized signals and potential gradients, we did the following experiments:

1) We increased the proportion of inducible cells in coculture, thus decentralizing paracrine signaling and coordination. The fraction of inducible cells in cocultures was inversely proportional to extrusion efficiency (Figure 7A),

2) We disrupted spatially organized signaling by flooding cocultures with saturating amounts of AREG. Exogenous AREG abolished directed migration and extrusion (Figure 7B-C).

Regarding the question about polarized structures forming as a result of signaling gradients, live-actin imaging experiments performed for a separate reviewer request were illuminating (see reviewer 1, point 2). In oncogenic cocultures, the Utrophin-261-EGFP reporter revealed polarized actin enrichment specifically at the leading edge of WT cells adjacent to oncogenic cells (Figure 5—figure supplement 2C-D). However, in the presence of EGFR inhibitor, polarized actin-containing basal protrusions were absent. This is a straightforward example of a polarized structure dependent on growth-factor signaling.

[Editors’ note: what follows is the authors’ response to the second round of review.]

Reviewer #1

Several articles have recently highlighted differential outcomes of pulsatile vs sustained ERK signalling and their relevance to homeostasis versus transformation, making this addition to the field timely. We feel that the authors have greatly improved their data since the last submission and are in favour of its acceptance, once they address the following concerns.

1) The Abstract describes the work in a very diffuse way, not concisely stating what the results are. One should be able to understand what the take-home message of the paper is saying from the Abstract. I think that the Nature website gives a good guide on how to write a useful Abstract. It is important to note that most people will only ever read this. Relay exactly what your paper's results are, i.e. exactly what you find in Figure 8.

We have now rewritten the Abstract to describe the findings in a more clear and direct way.

2) The same is true in your Discussion. The first paragraph should be a nice recap of your findings that points to Figure 8. Instead that first paragraph is vague and difficult to follow. The last paragraph should be more of a zoom out to talk about the impact of this work in general, instead of just repeating what you have said several times.

We have now restructured the Discussion as requested.

3) The authors have addressed the previous concerns by adding a substantial amount of data, but some quantifications (e.g. radial histograms in Figures 5, 6, 7) still lack statistical analysis.

Thanks. We have now added the missing statistics to all panels.
